# Nature and Strength of Lewis Acid/Base Interaction in Boron and Nitrogen Trihalides

**DOI:** 10.1002/asia.202001127

**Published:** 2020-10-21

**Authors:** Daniela Rodrigues Silva, Lucas de Azevedo Santos, Matheus P. Freitas, Célia Fonseca Guerra, Trevor A. Hamlin

**Affiliations:** ^1^ Department of Theoretical Chemistry Amsterdam Institute of Molecular and Life Sciences (AIMMS) Amsterdam Center for Multiscale Modeling (ACMM) Vrije Universiteit Amsterdam De Boelelaan 1083 1081 HV Amsterdam (The Netherlands; ^2^ Departamento de Química Universidade Federal de Lavras 37200-900 Lavras–MG Brazil; ^3^ Leiden Institute of Chemistry Gorlaeus Laboratories Leiden University Einsteinweg 55 2333 CC Leiden (The Netherlands

**Keywords:** Activation strain analysis, Bond energy, Density functional calculations, Energy decomposition analysis, Lewis acid-base pairs

## Abstract

We have quantum chemically investigated the bonding between archetypical Lewis acids and bases. Our state‐of‐the‐art computations on the X_3_B−NY_3_ Lewis pairs have revealed the origin behind the systematic increase in B−N bond strength as X and Y are varied from F to Cl, Br, I, H. For H_3_B−NY_3_, the bonding trend is driven by the commonly accepted mechanism of donor−acceptor [HOMO(base)−LUMO(acid)] interaction. Interestingly, for X_3_B−NH_3_, the bonding mechanism is determined by the energy required to deform the BX_3_ to the pyramidal geometry it adopts in the adduct. Thus, Lewis acids that can more easily pyramidalize form stronger bonds with Lewis bases. The decrease in the strain energy of pyramidalization on going from BF_3_ to BI_3_ is directly caused by the weakening of the B−X bond strength, which stems primarily from the bonding in the plane of the molecule (σ‐like) and not in the π system, at variance with the currently accepted mechanism.

## Graphical abstract

**That's strainful**! The stability of Lewis pairs involving boron trihalides and ammonia is driven by the energy required to deform the Lewis acids upon complexation, while the stability of Lewis pairs between borane and nitrogen trihalides is determined by the strength of orbital interactions.

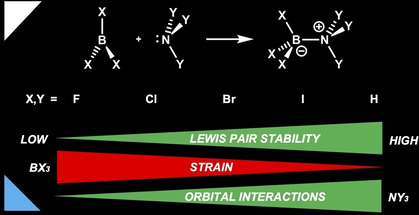

## Introduction

The chemistry of Lewis acids and bases is rich and can be found in any general chemistry textbook.^[1]^ In his epochal work,^[2]^ Gilbert N. Lewis introduced the concept of electron‐pair donor‐acceptor complexes, on which the current understanding of Lewis acid/base interactions is based. It defines Lewis acids as chemical species that accepts an electron‐pair from a Lewis base to form a Lewis adduct. Thus, the Lewis acidity and basicity scales are associated with the stability of the adducts, that is, relative to a reference, a stronger Lewis acid or Lewis base forms a stronger bounded Lewis complex. The Lewis acid/base chemistry has experienced continuous development since then^[3]^ and has found utility in a wide range of research areas, including catalysis^[4]^ and the recent advent of frustrated Lewis pair chemistry,^[5]^ to name a few.

Due to the ubiquity of Lewis acid/base in chemistry, attempts to rationalize the nature and strength of this interaction abound.^[6]^ The theory of hard and soft acids and bases (HSAB) proposed by Pearson^[7]^ is undoubtedly the most popular qualitative model used to understand this interaction. The HSAB principle uses the intrinsic properties of the interacting species to explain the stability of acid/base complexes, namely, the concept of hardness and softness, which is based on properties such as size, polarizability, and electronegativity. In this model, a hard base (the term “hard” stands for small sized atoms with low polarizability and high electronegativity) would preferentially bind to a hard acid, while a soft base (the term “soft” stands for large sized atoms with high polarizability and low electronegativity) prefers to associate with a soft acid. However, the validity of this model has been questioned, as it has been shown to fail in predicting reactivity of archetypal reactions.^[8]^

Interestingly, the relative Lewis acidity of boron trihalides with respect to strong bases (*e. g*., NH_3_, NMe_3_) is known to increase along the series BF_3_<BCl_3_<BBr_3_; however, the opposite trend is observed for the interaction with weak bases (*e. g*., N_2_, CH_3_F).[^6i^, ^9^] This indicates that Lewis acid/base is a rather complex interaction that depends on the entire system, not only on the characteristics of the isolated acids and bases. Over the years, various theories have been proposed to explain the trends in stability of Lewis pairs involving boron trihalides, such as those based on π‐backdonation,[^9c^, ^10^] the ability to engage in stabilizing orbital interactions^[11]^ or electrostatics,^[9a]^ ligand close packing (LCP) model,^[12]^ or electrophilicity principle.^[13]^ The decreased Lewis acidity of BF_3_ towards strong bases, compared to heavier boron trihalides, is widely attributed to a more efficient π charge donation from the fluorine lone‐pair into the empty p orbital of the boron (π‐backdonation), which reduces the availability of the boron atom to accept an electron pair from the Lewis base.[^9c^, ^10^] However, it has been shown that the p(π)‐p(π) overlap integral and the p(π) population at the boron is actually smaller for BF_3_ than for BCl_3_.[^11c^, ^14^] Alternatively, an intuitive argument based on the strength of frontier molecular orbital interactions has been proposed by Bessac and Frenking,^[11b]^ that is, the energy of the LUMO of BX_3_ decreases from X = F to Cl and results in more stabilizing orbital interactions with the HOMO of the Lewis base for BCl_3_ compared to BF_3_. We note that these explanations are universal and neither can explain the reversal in Lewis acidities that is observed for the Lewis complexes between boron trihalides and weak bases.

**Scheme 1:**
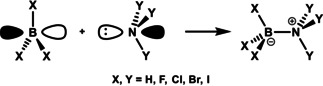
Formation of the Lewis pairs analyzed in this work.

We aim to illuminate the nature and strength of Lewis acid/base interaction within the conceptual framework provided by Kohn−Sham molecular orbital (KS‐MO) theory and ultimately provide a unified framework to understand Lewis pairs. To this end, we investigate the underlying physical mechanism behind the formation of a systematic set of X_3_B−NY_3_ Lewis pairs (Scheme 1, where X,Y = H, F, Cl, Br, and I). We first explore the archetypical borane−ammonia adduct, H_3_B−NH_3_, and then separately evaluate the substituent effect on the Lewis acid and Lewis base by varying X,Y from H to F, Cl, Br, and I. To the best of our knowledge, this is the first thorough analysis on the formation of Lewis pairs involving the complete series of nitrogen and boron trihalides. Detailed analysis of the electronic structures and bonding mechanisms enable us to interpret our results in quantitative and chemically meaningful terms, which reveals the role of different components, namely, charge‐transfer, electrostatic interaction and also strain energy, in the stability of the Lewis complexes. This demonstrates that, similar to hydrogen bonds,^[15]^ Lewis acid/base interaction is a complex interplay of several energy components, whose importance depends on the molecular system and may not be easily captured in simple predictive models.

## Methods

### Computational Details

All calculations were performed using the Amsterdam Density Functional (ADF) software package.^[16]^ Geometries and energies were calculated at the BLYP level of the generalized gradient approximation (GGA); exchange functional developed by Becke (B), and the GGA correlation functional developed by Lee, Yang and Parr (LYP).^[17]^ The DFT‐D3(BJ) method developed by Grimme and coworkers,^[18]^ which contains the damping function proposed by Becke and Johnson,^[19]^ was used to describe non‐local dispersion interactions. Scalar relativistic effects are accounted for using the zeroth‐order regular approximation (ZORA).^[20]^ Molecular orbitals (MO) were expanded in a large uncontracted set of Slater type orbitals (STOs) containing diffuse functions: TZ2P.^[21]^ The basis set is of triple‐ξ quality for all atoms and has been augmented with two sets of polarization functions. All electrons were included in the variational process, *i. e*., no frozen core approximation was applied. An auxiliary set of s, p, d, f, and g STOs was used to fit the molecular density and to represent the Coulomb and exchange potentials accurately in each self‐consistent field cycle. The accuracies of the fit scheme (ZLM fit)^[22]^ and the integration grid (Becke grid)^[23]^ were set to ‘very good’. The Lewis acids were optimized with *D*_3h_ symmetry constraints, and the Lewis bases and Lewis adducts were optimized with *C*_3v_ symmetry constraints. All optimized structures were confirmed to be true minima through vibrational analyses^[24]^ (no imaginary frequencies). The molecular structures were illustrated using CYLview.^[25]^

### Activation Strain and Energy Decomposition Analysis

Insight into the nature of Lewis acid/base interaction is obtained by applying the activation strain model (ASM)^[26]^ along the formation of the Lewis adducts. The formation of the Lewis pairs is computationally modelled by decreasing the distance between the boron atom of the Lewis acid and the nitrogen atom of the Lewis base, while other geometry parameters are included in the optimization. Thus, each analysis starts from an optimized Lewis acid and Lewis base at a relatively large distance, then, the B−N distance (r_B−N_) is gradually decreased to a bond length smaller than the equilibrium distance of the Lewis adduct.

The activation strain model of chemical reactivity^[26]^ is a fragment‐based approach to understand the energy profile of a chemical process in terms of the original reactants (*i. e*., the formation of the dimer from monomers). Thus, the overall bond energy Δ*E*(ξ) is decomposed into the respective total strain and interaction energy, Δ*E*_strain_(ξ) and Δ*E*_int_(ξ), and project these values onto the reaction coordinate ξ (in this case, r_B−N_) [Eq. 1].

(1) $$\Delta E\left(\xi \right)=\Delta E{}_{\mathrm{strain}}\left(\xi \right)+\Delta E{}_{\mathrm{int}}\left(\xi \right)$$

In this equation, the total strain energy Δ*E*_strain_(ξ) is the penalty that needs to be paid to deform the reactants from their equilibrium structure to the geometry they adopt in the complex at point ξ of the reaction coordinate. On the other hand, the interaction energy Δ*E*_int_(ξ) accounts for all the chemical interactions that occur between the deformed fragments along the reaction coordinate. The total strain energy can, in turn, be further decomposed into the strain energies corresponding to the deformation of the Lewis acid $\Delta E_{\mathrm{strain}{,\mathrm{BX}}_{3}}\left(\xi \right)$ as well as from the Lewis base $\Delta E_{\mathrm{strain}{,\mathrm{NY}}_{3}}\left(\xi \right)$ [Eq. 2].

(2) $$\Delta E_{\mathrm{strain}}\left(\xi \right)=\Delta E_{\mathrm{strain}{,\mathrm{BX}}_{3}}\left(\xi \right)+\Delta E_{\mathrm{strain}{,\mathrm{NY}}_{3}}\left(\xi \right)$$

The interaction energy between the deformed fragments is further analyzed in terms of quantitative Kohn−Sham molecular orbital (KS‐MO) theory in combination with a canonical energy decomposition analysis (EDA).^[27]^ The EDA decomposes the Δ*E*_int_(ξ) into the following four physically meaningful energy terms [Eq. 3]:

(3) $$\Delta E{}_{\mathrm{int}}\left(\xi \right)\;=\;\Delta V{}_{\mathrm{elstat}}\left(\xi \right)+\Delta E{}_{\mathrm{Pauli}}\left(\xi \right)+\Delta E{}_{\mathrm{oi}}\left(\xi \right)+\Delta E{}_{\mathrm{disp}}\left(\xi \right)$$

Herein, Δ*V*_elstat_(ξ) is the classical electrostatic interaction between the unperturbed charge distributions of the (deformed) fragments and is usually attractive. The Pauli repulsion Δ*E*_Pauli_(ξ) comprises the destabilizing interaction between occupied closed‐shell orbitals of both fragments due to the Pauli principle. The orbital interaction energy Δ*E*_oi_(ξ) accounts for polarization and charge transfer between the fragments, such as HOMO−LUMO interactions. It can be decomposed into the contributions from each irreducible representation Γ of the interacting system [Eq. (4)]. Finally, the dispersion energy Δ*E*_disp_(ξ) accounts for the dispersion corrections as introduced by Grimme et al.^[18]^ A detailed, step‐by‐step, guide on how to perform and interpret the ASM and EDA can be found in reference 26a. The Pyfrag program was used to facilitate these analyses.^[28]^

(4) $$\Delta E_{\mathrm{oi}}=\sum_{\Gamma }\Delta E_{\Gamma }$$

### Voronoi Deformation Density (VDD) charges

The atomic charge distribution was analyzed by using the Voronoi Deformation Density (VDD) method.^[29]^ The VDD method partitions the space into so‐called Voronoi cells, which are non‐overlapping regions of space that are closer to nucleus A than to any other nucleus. The charge distribution is determined by taking a fictitious promolecule as reference point, in which the electron density is simply the superposition of the spherical atomic densities. The change in density in the Voronoi cell when going from this promolecule to the final molecular density of the interacting system is associated with the VDD atomic charge *Q*. Thus, the VDD atomic charge *Q*_A_^VDD^ of atom A is given by:

(5) $$Q_{A}^{\mathrm{VDD}}=-\int_{\mathrm{Voronoi}\;\mathrm{cell}\;\mathrm{of}\;A}[\rho \left(r\right)-\rho_{promolecule}\left(r\right)]\mathrm{dr}$$

Instead of computing the amount of charge contained in an atomic volume, we compute the flow of charge from one atom to the other upon formation of the molecule. The physical interpretation is therefore straightforward. A positive atomic charge *Q*_A_ corresponds to the loss of electrons, whereas a negative atomic charge *Q*_A_ is associated with the gain of electrons in the Voronoi cell of atom A.

## Results and Discussion

### Structures and bond strengths

**Figure 1:**
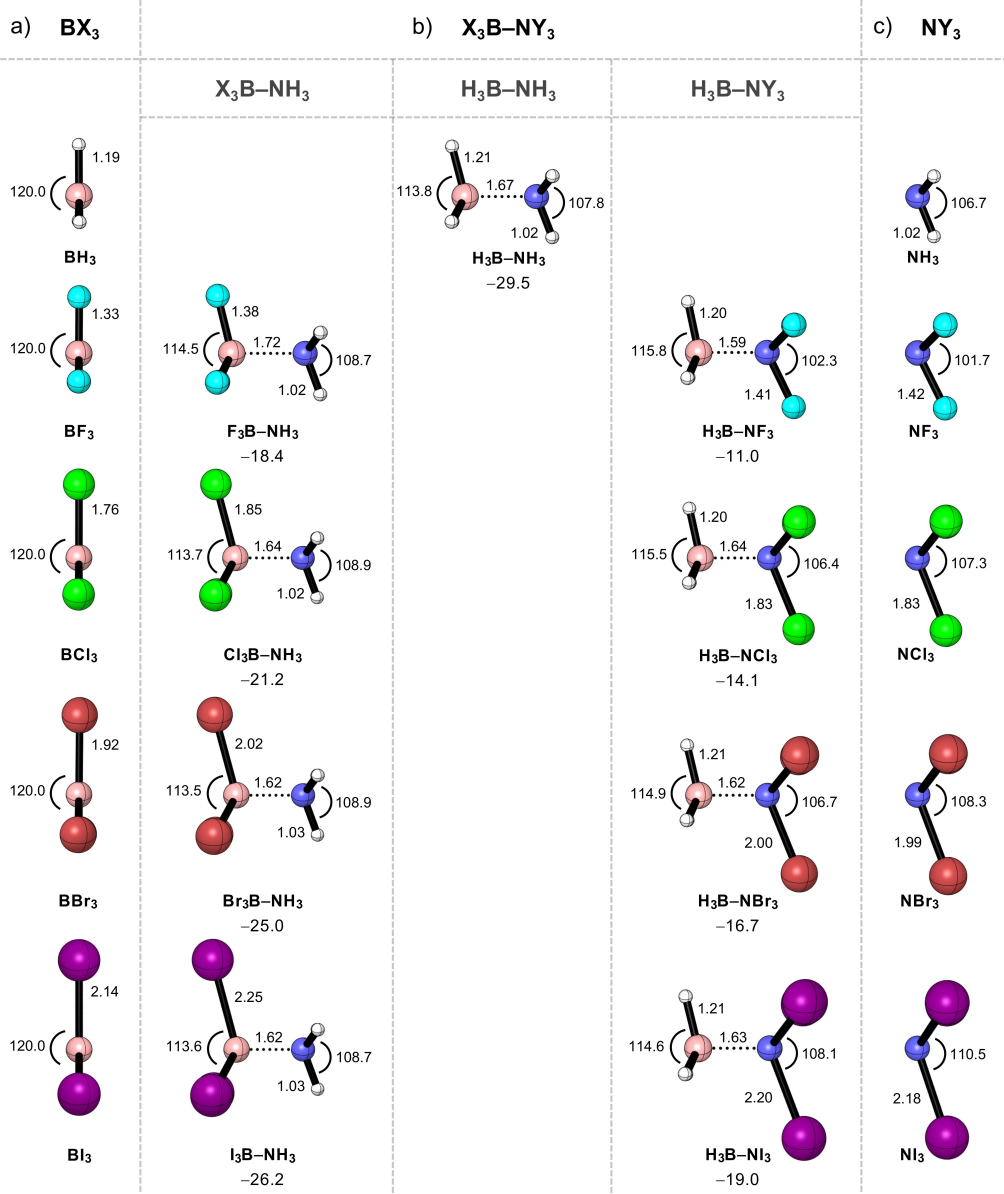
Equilibrium geometries (in Å, deg.) of the a) BX_3_ Lewis acids, b) X_3_B−NY_3_ Lewis adducts, and c) NY_3_ Lewis bases (X,Y = H, F, Cl, Br, and I), along with the electronic bond energies Δ*E* (in kcal mol^−1^) computed at ZORA‐BLYP‐D3(BJ)/TZ2P.

In this section, the geometries and bond energies of the X_3_B−NY_3_ Lewis pairs (X,Y = H, F, Cl, Br, and I) are discussed. The results are summarized in Figure 1 (full structural data is provided in Table S1 in the Supporting Information). As BX_3_ and NY_3_ approach each other to form the Lewis adduct, the Lewis acid must pyramidalize from its trigonal planar equilibrium geometry, that is, the θ_X−B−X_ angle decreases and the r_B−X_ bond length increases (Table S1). This effect is much less pronounced in the Lewis base, as it already has a pyramidal equilibrium geometry and undergoes almost no deformation upon complexation. Our computed bond lengths and angles of borane−ammonia (*i. e*., H_3_B−NH_3_) are in very good agreement with existing experimental data^[30]^ (in parenthesis): r_B−N_ bond length of 1.675 Å (1.657 Å), r_B−H_ bond length of 1.211 Å (1.216 Å), r_N−H_ bond length of 1.022 Å (1.014 Å), θ_H−B−H_ angle of 113.8° (113.8°), and θ_H−N−H_ angle of 107.8° (108.7°).

The expected trends in Lewis adduct stabilities are nicely reproduced by our DFT computations at ZORA‐BLYP‐D3(BJ)/TZ2P. Borane−ammonia forms the strongest bond complex in our series of Lewis pairs (Δ*E* = −29.5 kcal mol^−1^). Upon substitution of the hydrogen atoms on the Lewis acid or Lewis base with halogen atoms, the energy of formation of the Lewis adduct Δ*E* decreases in strength, *i. e*., becomes less stabilizing, along the series: H, I, Br, Cl, F. The bond enthalpies at 298 K (Δ*H*_298.15_) show the same trends as the electronic bond energies Δ*E* (see supporting methods and Table S1 in the Supporting Information). In the following sections, we partition the Lewis pairs into three sets: 1) H_3_B−NH_3_, 2) X_3_B−NH_3_, and 3) H_3_B−NY_3_ (where X,Y = F, Cl, Br, and I), and provide a unified model to rationalize the strength of the Lewis pair bond through detailed analyses of the electronic structure and bonding mechanism.

### Borane‐Ammonia

**Figure 2:**
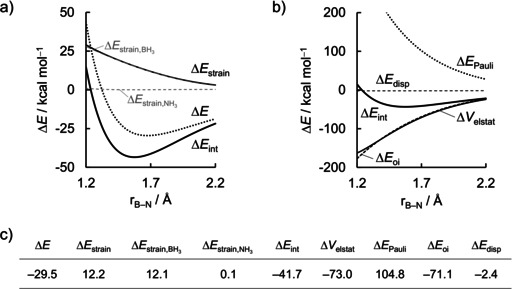
a) Activation strain model and b) energy decomposition analysis of the H_3_B−NH_3_ Lewis pair projected onto the forming B−N bond distance, and c) data (in kcal mol^−1^) at consistent geometry with a B−N distance of 1.687 Å. Computed at ZORA‐BLYP‐D3(BJ)/TZ2P.

The activation strain model and energy decomposition analysis diagrams of the borane−ammonia adduct are shown in Figure 2. From Figure 2a, it can be easily seen that the energy profile in Δ*E* curve along the newly forming B−N bond is determined by the interaction energy Δ*E*_int_, which becomes destabilizing only at very short B−N bond distance (smaller than r_B−N_<1.230 Å). The strain energy Δ*E*_strain_, on the other hand, becomes increasingly destabilizing as the internuclear distance decreases. The destabilizing Δ*E*_strain_ stems mostly from the deformation of the Lewis acid, BH_3_, from its planar equilibrium geometry to the pyramidal geometry it adopts in the complex. Note that the BH_3_ strain energy curve $\Delta E_{\mathrm{strain}{,\mathrm{BH}}_{3}}$ coincides with the total strain energy curve Δ*E*_strain_, whereas the NH_3_ strain energy curve $\Delta E_{\mathrm{strain}{,\mathrm{NH}}_{3}}$ is flat all along the reaction coordinate.

Since the interaction energy plays a critical role on the formation of the H_3_B−NH_3_ Lewis pair, we further decomposed Δ*E*_int_ into four physically meaningful terms according to Eq. (3). The results of this energy decomposition analysis (EDA) are shown in Figure 2b. This graph shows us a quite straightforward picture. The Δ*E*_int_ is equally stabilized by orbital and electrostatic interactions, the Δ*E*_oi_ and Δ*V*_elstat_ curves nearly coincide at all B−N bond distances shown. Both terms become more stabilizing as the fragment separation decreases and the bond begins to form, because of the increase in both HOMO−LUMO orbital overlap and charge penetration of nuclei with electron clouds. The stabilizing effect of Δ*E*_oi_ and Δ*V*_elstat_ is, however, opposed by the Pauli repulsion Δ*E*_Pauli_ term. Note that at a B−N separation shorter than the equilibrium bond length, the upward slope of the Δ*E*_Pauli_ curve is larger than the downward slope of the Δ*E*_oi_ and Δ*V*_elstat_ curves, which is the reason behind the destabilization of Δ*E*_int_ at short internuclear distance.^[31]^ The dispersion energy Δ*E*_disp_, on the other hand, remains nearly constant at any point along r_B−N_.

Thus, electrostatic and orbital interactions are the main contributors to the formation of the H_3_B−NH_3_ Lewis pair. To understand the origin of the stabilizing Δ*E*_oi_ and Δ*V*_elstat_, we have analyzed the molecular orbital (MO) diagram of the fragment molecular orbitals (FMOs) and the electrostatic potential surface of each fragment, respectively.^[26a]^ To ensure that our results are not skewed by the fact that the Lewis adducts have different equilibrium bond lengths, analysis of all Lewis pairs will be performed at the same r_B−N_ distance of 1.687 Å, near to the equilibrium bond distance of borane−ammonia. Energies at consistent geometry for the H_3_B−NH_3_ adduct are shown in Figure 2c.

**Figure 3:**
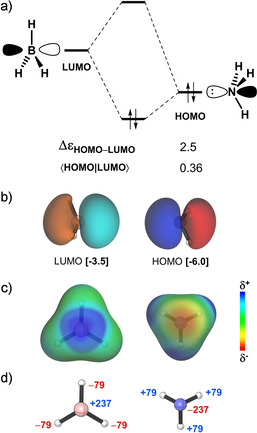
a) MO diagram along with the orbital energy gap (in eV) and overlap of the HOMO(base)−LUMO(acid) interaction in the H_3_B−NH_3_ Lewis pair, b) isosurface (at 0.05 au) and energy (in eV) of the HOMO and LUMO orbitals of the a_1_ irreducible representation of the *C*_3*v*_ symmetry, c) electrostatic potential surfaces (at 0.01 au) from −0.1 (red) to 0.1 (blue) au and d) VDD atomic charges (in milli‐electrons). Computed at consistent geometry with a B−N bond distance of 1.687 Å at ZORA‐BLYP‐D3(BJ)/TZ2P.

Figure 3a shows that Δ*E*_oi_ can be rationalized in terms of the well‐known [HOMO(base)−LUMO(acid)] interaction between the filled N 2p_z_ orbital of the Lewis base with the empty B 2p_z_ orbital of the Lewis acid (see Figure 3b). This interaction has favorable orbital energy gap (Δϵ = 2.5 eV) and overlap (⟨HOMO|LUMO⟩ = 0.36).^[32]^ Furthermore, inspection of the electrostatic potential surfaces illustrated in Figure 3c and atomic charges in Figure 3d reveals that accumulation of positive charge around the boron atom of the electron‐deficient Lewis acid and negative charge around the nitrogen atom of the electron‐rich Lewis base are responsible for the stabilizing Δ*V*_elstat_.

In summary, the EDA along the forming H_3_B−NH_3_ Lewis pair demonstrates that the attractive interaction between the BH_3_ Lewis acid and the NH_3_ Lewis base has a stabilizing covalent character that is the same magnitude as the electrostatic character, both can be easily understood in terms of simple chemical arguments. Our results, so far, conform to and agree with the current picture presented in the literature.[^6d^, ^6e^, ^6i^] In the coming next sections, we extend our analysis to study the stability of Lewis adducts of halogenated Lewis acids and Lewis bases.

### Halogenated Lewis Acids

**Figure 4:**
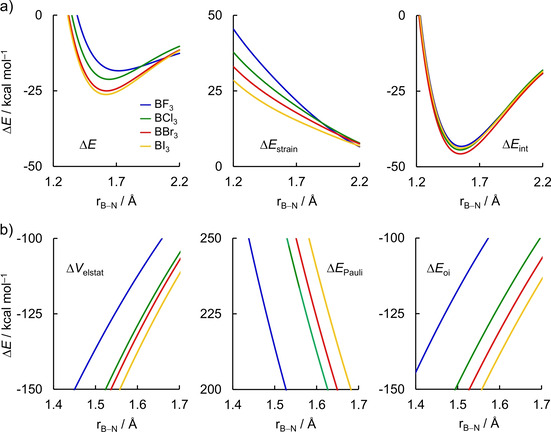
a) Activation strain model and b) energy decomposition analysis of the X_3_B−NH_3_ Lewis pairs projected onto the forming B−N bond distance (where X = F, Cl, Br, and I) computed at ZORA‐BLYP‐D3(BJ)/TZ2P. Dispersion energy Δ*E*_disp_ not shown, see Table S2 for data at consistent geometries.

Next, we turn to the analysis of the formation of the Lewis pairs between boron trihalides and ammonia. The activation strain model and energy decomposition analysis diagrams for the X_3_B−NH_3_ Lewis pairs (where X = F, Cl, Br, and I) are shown in Figure 4. In line with the expected Lewis acidities,^[9c]^ BI_3_ forms the strongest complex with ammonia and the energy of formation of the Lewis adduct Δ*E* decreases in strength, *i. e*., becomes less stabilizing, along the series: BI_3_, BBr_3_, BCl_3_, BF_3_. However, in contrast with the commonly accepted view of Lewis acid/base interaction, the stronger bond energy does not originate from the more stabilizing interaction energy, but from the *less destabilizing strain energy*.[^12a^, ^33^] In general, Δ*E*_strain_ is less destabilizing for the Lewis complex involving BI_3_ and becomes increasingly destabilizing along the series BI_3_<BBr_3_<BCl_3_<BF_3_. On the other hand, Δ*E*_int_ is nearly the same for all Lewis adducts and does not follow a systematic trend. If covalent interactions would be the decisive factor for the observed Lewis pair stabilities, one would expect that the trend in Δ*E*_int_ along the boron trihalides also hold for the trend in Δ*E*; but this is not the case. We discuss these findings in more details below.

The same conclusion can be drawn at consistent geometries (r_B−N_ = 1.687 Å, see Table 1; the EDA data is given in Table S2). The values of Δ*E*_int_ are of the same order of magnitude as in borane−ammonia, *ca*. 41 kcal mol^−1^, while the Δ*E*_strain_ is significantly larger for the boron trihalides and accounts for 12.2 and 22.3 kcal mol^−1^ for H_3_B−NH_3_ and F_3_B−NH_3_, respectively. The Δ*E*_strain_ results predominantly from the deformation of the Lewis acid $\Delta E_{\mathrm{strain}{,\mathrm{BX}}_{3}}$ . Nevertheless, there is no clear correlation of $\Delta E_{\mathrm{strain}{,\mathrm{BX}}_{3}}$ with any geometrical change. The pyramidalization angle ${\Delta \theta }_{\mathrm{pyr}{,\mathrm{BX}}_{3}}$ is very similar for all Lewis acids and the B−X bond stretching Δr_B−X_ has a reversed trend that from $\Delta E_{\mathrm{strain}{,\mathrm{BX}}_{3}}$ , *i. e*., the Δr_B−X_ increases as X goes from F to I (see Table 1).

**Table 1:** Activation strain model terms (in kcal mol^−1^), bond stretching (in Å) and pyramidalization angle (in degrees) computed at consistent geometries with a B−N distance of 1.687 Å of the X_3_B−NH_3_ Lewis pairs (where X = F, Cl, Br, and I).^[a]^

Lewis acid	Δr_B−X_	Δr_N−H_	Δθ_pyr,BX3_^[b]^	Δθ_pyr,NH3_^[b]^	Δ*E*	Δ*E*_int_	Δ*E*_strain_	$\Delta E_{\mathrm{strain}{,\mathrm{BX}}_{3}}$
BF_3_	0.060	0.001	−17.2	5.7	−18.3	−40.6	22.3	22.2
BCl_3_	0.089	0.002	−18.0	7.4	−21.0	−41.3	20.3	20.1
BBr_3_	0.096	0.003	−18.0	7.7	−24.5	−42.6	18.1	17.8
BI_3_	0.103	0.004	−17.6	7.5	−25.6	−41.2	15.5	15.3

[a] Computed at ZORA‐BLYP‐D3(BJ)/TZ2P, geometrical data relative to the separate reactants. [b] Pyramidalization angle defined as the sum of the three θ_X−B−X_ and θ_H−N−H_ angles for BX_3_ and NH_3_, respectively.

In order to pinpoint the origin of the observed strain energy of the boron trihalides, we have carried out a subsequent analysis on the BX_3_ fragment. This time we decompose the $\Delta E_{\mathrm{strain}{,\mathrm{BX}}_{3}}$ term into the individual strain energies associated with the bending of the θ_X−B−X_ angle (Δ*E*_strain,θ_) and the B−X bond stretch (Δ*E*_strain,r_), as schematically illustrated in Table 2. First, the BX_3_ is pyramidalized with a fixed r_B−X_, taken from the respective planar equilibrium geometry, and, next, the r_B−X_ bond is allowed to relax to the one it has in the consistent geometry of the Lewis pair. The energy associated with each geometrical deformation is presented in Table 2. The majority of the strain energy originates from the bending of the θ_X−B−X_ angle and the trends in Δ*E*_strain,θ_ follow exactly the trends of the total strain of the Lewis acid $\Delta E_{\mathrm{strain}{,\mathrm{BX}}_{3}}$ , that is, it is larger for BF_3_ and smaller for BI_3_. The same trend can be observed if we analyze the other way around, first elongation of the r_B−X_ bond and then bending of the θ_X−B−X_ angle (see Figure S2 and Table S3). Yet, the pyramidalization angle is similar for all boron trihalides. Why then does BX_3_ become easier to bend to the same extent along the series X = F, Cl, Br, I?

**Table 2:** The strain energy terms (in kcal mol^−1^) associated with the step‐by‐step deformation of the Lewis acid from the planar to the pyramidal geometry.^[a]^

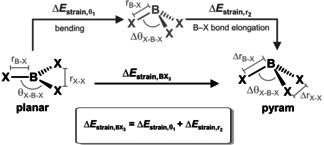			
Lewis acid	$\Delta E_{\mathrm{strain}{,\mathrm{BX}}_{3}}$	$\Delta E_{\mathrm{strain}{,\theta }_{1}}$	$\Delta E_{\mathrm{strain}{,r}_{2}}$
BF_3_	22.2	19.1	3.1
BCl_3_	20.1	16.7	3.4
BBr_3_	17.8	14.9	2.9
BI_3_	15.3	12.8	2.5

[a] Geometry adopted in the complex with a B−N distance of 1.687 Å of the X_3_B−NH_3_ Lewis pairs (where X = F, Cl, Br, and I), computed at ZORA‐BLYP‐D3(BJ)/TZ2P.

To answer to this question, we must understand exactly how the electronic structure of the Lewis acid changes upon pyramidalization (that is, bending and elongation). The rise in energy associated with the deformation of BX_3_ (*i. e*., $\Delta E_{\mathrm{strain}{,\mathrm{BX}}_{3}}$ ) could stem from two distinct factors: i) the bonding between central boron and halogen ligands becomes less stabilizing in the pyramidal geometry; and ii) there is an increase in the repulsion among the halogens as BX_3_ deforms.^[34]^ Therefore, we have further decomposed the $\Delta E_{\mathrm{strain}{,\mathrm{BX}}_{3}}$ in terms of the interaction energy between B and X_3_ ($\Delta E_{\mathrm{int}{,B-X}_{3}}$ ) and among the three X (Δ*E*_int,X−X−X_), more specifically, in terms of the change in both energy terms as BX_3_ deforms from the planar to the pyramidal geometry (Table 3; see supporting methods for a complete derivation).

**Table 3:** Change in the energy decomposition analysis terms (in kcal mol^−1^) associated with the deformation of the BX_3_ Lewis acids from the planar to the pyramidal geometry^[a]^ (where X = F, Cl, Br, and I).^[b]^

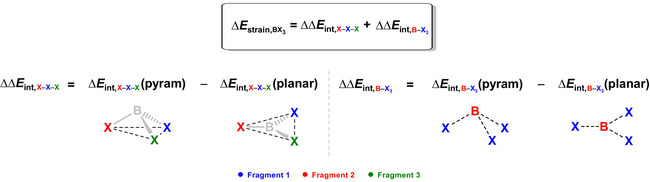									
Lewis acid	$\Delta E_{\mathrm{strain}{,\mathrm{BX}}_{3}}$	ΔΔ*E*_int,X−X−X_	$\Delta \Delta E_{\mathrm{int}{,B-X}_{3}}$	$\Delta \Delta E_{\mathrm{elstat}{,B-X}_{3}}$	$\Delta \Delta E_{\mathrm{Pauli}{,B-X}_{3}}$	$\Delta \Delta E_{\mathrm{oi}{,B-X}_{3}}$	$\Delta \Delta E_{\mathrm{oi}{,a}_{1}}$	$\Delta \Delta E_{\mathrm{oi}{,e}_{1}}$	$\Delta \Delta E_{\mathrm{oi}{,a}_{2}}$
BF_3_	22.2	−4.8	27.0	74.7	−142.5	94.7	27.1	67.1	0.5
BCl_3_	20.1	−6.5	26.6	95.8	−126.0	56.8	13.5	43.0	0.3
BBr_3_	17.8	−5.7	23.5	102.5	−122.0	43.1	8.5	34.4	0.2
BI_3_	15.3	−5.1	20.4	99.3	−112.1	33.2	5.8	27.3	0.1

[a] Geometry adopted in the complex with a B−N distance of 1.687 Å of the X_3_B−NH_3_ Lewis pairs. [b] Computed at ZORA‐BLYP‐D3(BJ)/TZ2P.

Put simply, the interaction energy Δ*E*_int,X−X−X_ corresponds to the formation of the (X^.^)_3_ fragment in its quartet valence configuration and in the geometry which it acquires in the overall molecule, and the interaction energy $\Delta E_{\mathrm{int}{,B-X}_{3}}$ corresponds to the actual energy change when the prepared B‐sp^2^ and (X^.^)_3_ fragments are combined to form the BX_3_ (planar or pyramidal). As BX_3_ goes from one geometry to the other, the change in interaction energy is written as ΔΔ*E*_int_. Thus, the ΔΔ*E*_int,X−X−X_ and $\Delta \Delta E_{\mathrm{int}{,B-X}_{3}}$ are, respectively, the change in both interaction energy terms when BX_3_ goes from the planar to the pyramidal geometry and sum to $\Delta E_{\mathrm{strain}{,\mathrm{BX}}_{3}}$ (see Table 3). Here, positive values of ΔΔ*E*_int_ indicate that the interaction energy opposes pyramidalization, while negative values indicate that it favors pyramidalization of the Lewis acid.

The most striking result in Table 3 is that the interaction energy between the halogens, which is predominantly repulsive (see Table S4), becomes less destabilizing in the pyramidal geometry (*i. e*., ΔΔ*E*_int,X−X−X_ is negative) and, thus, favors the pyramidalization of the Lewis acid. This is because when the r_B−X_ bond elongates, the halogens are actually farther removed from each other in the pyramidal than in the planar geometry (see Δr_X−X_ in Table S3). This means that $\Delta \Delta E_{\mathrm{int}{,B-X}_{3}}$ determines the trends in $\Delta E_{\mathrm{strain}{,\mathrm{BX}}_{3}}$ , as clearly observed from Table 3. Along X = F to I, $\Delta E_{\mathrm{strain}{,\mathrm{BX}}_{3}}$ varies from 22.2 to 15.3 kcal mol^−1^ and $\Delta \Delta E_{\mathrm{int}{,B-X}_{3}}$ varies from 27.0 to 20.4 kcal mol^−1^. The interaction energy between the boron and the halogens is less stabilizing in the pyramidal than in the planar geometry (*i. e*., $\Delta \Delta E_{\mathrm{int}{,B-X}_{3}}$ is positive) and, thus, opposes the pyramidalization of the Lewis acid. This loss in stabilization correlates to the difficulty to pyramidalize the Lewis acid, that is, a larger $\Delta \Delta E_{\mathrm{int}{,B-X}_{3}}$ translates in to a larger $\Delta E_{\mathrm{strain}{,\mathrm{BX}}_{3}}$ .

To obtain insight into the different contributors to the interaction energy we have again employed the EDA scheme^[27]^ (see Table 3, full data is provided in Table S4). It can be seen that the trend in $\Delta \Delta E_{\mathrm{int}{,B-X}_{3}}$ is dictated by the orbital interactions $\Delta \Delta E_{\mathrm{oi}{,B-X}_{3}}$ . Both $\Delta \Delta E_{\mathrm{int}{,B-X}_{3}}$ and $\Delta \Delta E_{\mathrm{oi}{,B-X}_{3}}$ oppose pyramidalization of the Lewis acid (*i. e*., they are positive) and decrease in magnitude from BF_3_ to BI_3_. Along X = F to I, $\Delta \Delta E_{\mathrm{oi}{,B-X}_{3}}$ varies from a value of 94.7 to 33.2 kcal mol^−1^. Note that the electrostatic interaction also opposes pyramidalization (*i. e*., positive values of $\Delta \Delta E_{\mathrm{elstat}{,B-X}_{3}}$ ) but it increases from BF_3_ to BI_3_, therefore, not following the trend in $\Delta \Delta E_{\mathrm{int}{,B-X}_{3}}$ . Interestingly, the Pauli repulsion term favors pyramidalization (*i. e*., negative values of $\Delta \Delta E_{\mathrm{Pauli}{,B-X}_{3}}$ ) because it goes with an elongation of the r_B−X_ bond in the pyramidal geometry, which becomes longer from BF_3_ to BI_3_. Therefore, in a sense, $\Delta \Delta E_{\mathrm{elstat}{,B-X}_{3}}$ and $\Delta \Delta E_{\mathrm{Pauli}{,B-X}_{3}}$ work together against the observed trend in $\Delta \Delta E_{\mathrm{int}{,B-X}_{3}}$ . Finally, the dispersion term, ΔΔ*E*_disp,B−X3_, is the same at both geometries (*i. e*., $\Delta \Delta E_{\mathrm{disp}{,B-X}_{3}}$ = 0.0 and is not provided in Table 3).

**Figure 5:**
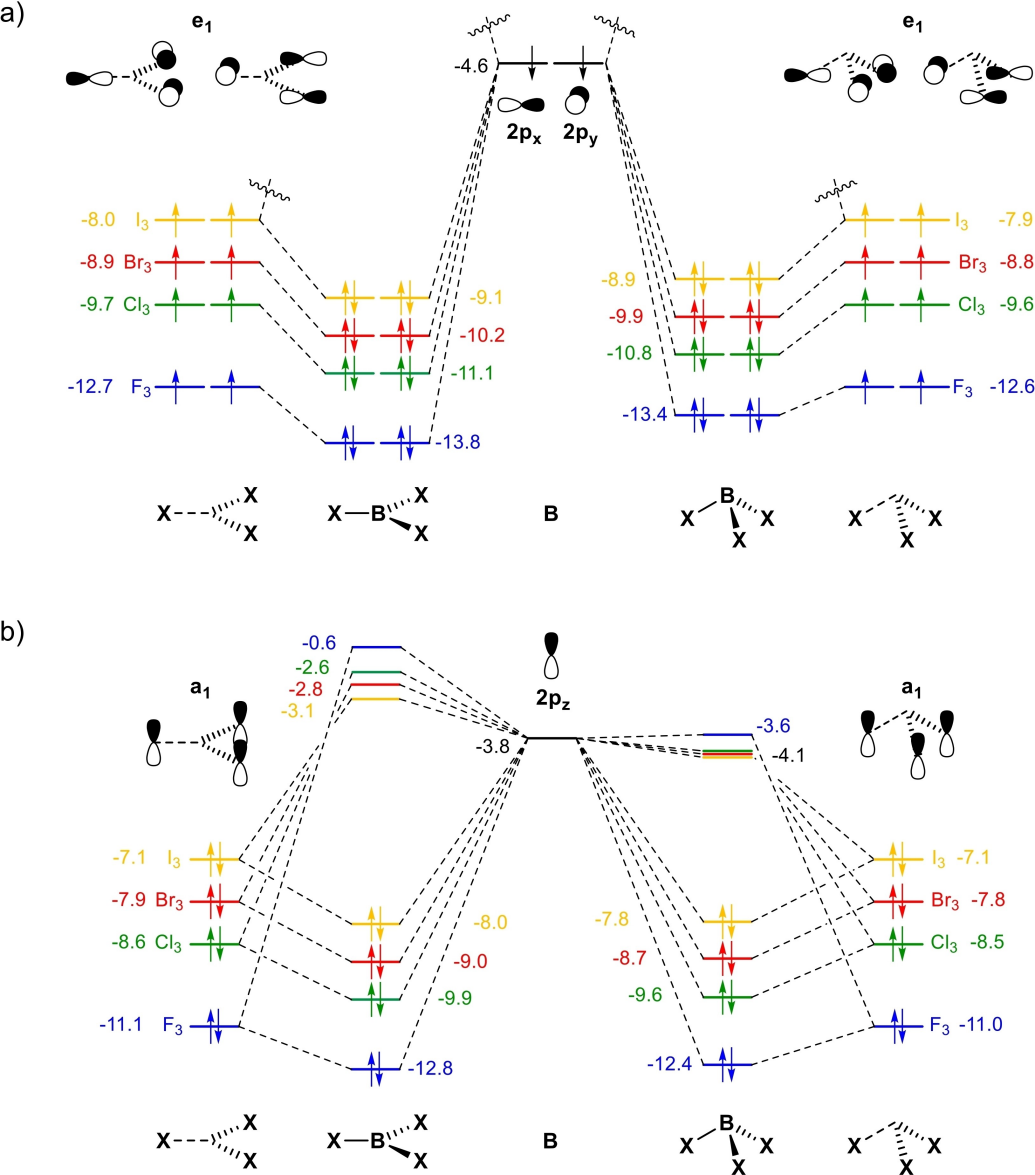
Orbital interaction scheme for planar and pyramidal BX_3_ (where X = F, Cl, Br, and I) in the a) e_1_ and b) a_1_ irreducible representations of the *C*_3*v*_ symmetry computed at ZORA‐BLYP‐D3(BJ)/TZ2P.

Figure 5 shows the MO diagram of the main orbital interactions between (X^.^)_3_ and B‐sp^2^ in the e_1_ and a_1_ representations (the complete MO diagram with all valence orbitals is provided in Figure S3). We now address why the covalent component of the interaction between B and X_3_ is less stabilizing in the pyramidal geometry, that is, $\Delta \Delta E_{\mathrm{oi}{,B-X}_{3}}$ is positive, and how it determines the trend in $\Delta \Delta E_{\mathrm{strain}{,\mathrm{BX}}_{3}}$ . Most of this effect originates from the orbital interactions in the e_1_ irreducible representation (see Table 3), which corresponds to the bonding in the plane of the molecule (σ‐like bonding). Interestingly, the total stabilizing orbital interactions $\Delta \Delta E_{\mathrm{oi}{,B-X}_{3}}$ is provided by nearly 70% $\Delta \Delta E_{\mathrm{oi}{,e}_{1}}$ and 30% $\Delta \Delta E_{\mathrm{oi}{,a}_{1}}$ (the contribution from $\Delta E_{\mathrm{oi}{,a}_{2}}$ is very small, see Table S4). This is in contrast to the common belief that the strength of the B−X bond arises from the overlap in the π system (*i. e*., in the a_1_ representation), between the filled *n*p_z_ orbitals of the halogens and the empty p_z_ orbital of boron.^[9c]^

In planar BX_3_ (Figure 5a left), two electron pair bonds are formed in the e_1_ irreducible representation (*n*e_1_±2p_x_ and *n*e_1_±2p_y_), where *n*e_1_ is a combination of the *n*p orbitals in the xy plane of the halogen atoms. The degenerated singly occupied *n*e_1_ orbitals show the well‐known increase in energy on descending group 17 in the periodic table,^[35]^ from −12.7 to −8.0 eV as X goes from F to I, associated with the decreasing electronegativity of X.^[36]^ As the fragments combine to form BX_3_, the electrons are stabilized in the bonding molecular orbitals and this stabilization correlates well with the energy of the (X^.^)_3_ fragment orbitals, in line with the order of strength of the B−X bond.^[37]^ Upon pyramidalization, there is a decrease in the orbital overlap between *n*e_1_ and 2p_x,y_ for all BX_3_ (see Table S4), resulting in the less stabilizing $\Delta \Delta E_{\mathrm{oi}{,B-X}_{3}}$ . Furthermore, pyramidalization also results in destabilization of the *n*e_1_ orbitals of the (X^.^)_3_ fragment and, most important, in the bonding molecular orbitals of BX_3_ (Figure 5a right). Interestingly, the destabilization of the bonding molecular orbitals shows the same trend as the $\Delta E_{\mathrm{strain}{,\mathrm{BX}}_{3}}$ , that is, it decreases along the series X = F, Cl, Br, I (Δϵ = 0.4, 0.3, 0.3, and 0.2 eV for BF_3_, BCl_3_, BBr_3_, and BI_3_, respectively). Similar effect occurs for orbital interactions in the a_1_ representation (see Figure 5b). Thus, as the boron trihalides deform to the same extent, the destabilization in the molecular orbitals of BF_3_ is larger. The (F^.^)_3_ is more strongly bound to the central boron atom, therefore, the decrease in the strain energy from BF_3_ to BI_3_ can be ascribed to the amount of energy required to distort a weaker bond. In other words, it requires less energy to deform BI_3_ than BF_3_ because the B−I bond is weaker than the B−F bond.

At last, we comment on the role of the orbital interactions between the Lewis acid and the Lewis base to the stability of the X_3_B−NH_3_ Lewis pairs (where X = F, Cl, Br, and I), which is the widely accepted rationale to explain the Lewis acidity of boron trihalides.^[11]^ Our EDA results (Figure 4b and Table S2), indeed, demonstrate that Δ*E*_oi_ follows the trend in Δ*E*, that is, it becomes more stabilizing from F_3_B−H_3_ to I_3_B−NH_3_. The trends in Δ*E*_oi_ can be ascribed to the energy of the LUMO of BX_3_ that decreases in energy from BF_3_ to BI_3_ (see Figure 5b), resulting in more stabilizing orbital interactions, in line with the results by Bessac and Frenking.^[11b]^ However, the stabilizing effect of Δ*E*_oi_ (and also Δ*V*_elstat_) is counteracted by a strong Pauli repulsion Δ*E*_Pauli_ that leads to a similar Δ*E*_int_ for all Lewis adducts (see Figure 4b). We again emphasize that it is crucial to compare the Lewis adducts at a consistent geometry, that is, the same r_B−N_ bond length, because the energy components are highly dependent on the bond distance.^[26a]^ Data at the equilibrium geometries (Table S1) shows that the strain energy of BF_3_ is smaller than BCl_3_, but this is just because of the longer r_B−N_ bond distance in the Lewis pair with the former. Analysis at the consistent geometries (Table 1) shows that the trends in bond energy Δ*E* can solely be assigned to the strain energy of the Lewis acid $\Delta E_{\mathrm{strain}{,\mathrm{BX}}_{3}}$ ; not to the interaction energy Δ*E*_int_.

We conclude that the more destabilizing strain energy along the series BI_3_<BBr_3_<BCl_3_<BF_3_, leads to less stable X_3_B−NH_3_ Lewis pairs (where X = F, Cl, Br, and I), due to a loss in stabilization of the bonding interactions between the central boron and the halogen ligands as the BX_3_ goes from the planar to the pyramidal geometry. This effect is most pronounced for BF_3_ because the B−F bond is the strongest in our series of boron trihalides. These general observations also explain why a reversed trend is observed for the interaction of boron trihalides with weak bases:^[9]^ weak bases induce small distortion of BX_3_ from its planar equilibrium geometry that allows the interaction energy to dominate and govern the bonding of these Lewis pairs.

### Halogenated Lewis Bases

**Figure 6:**
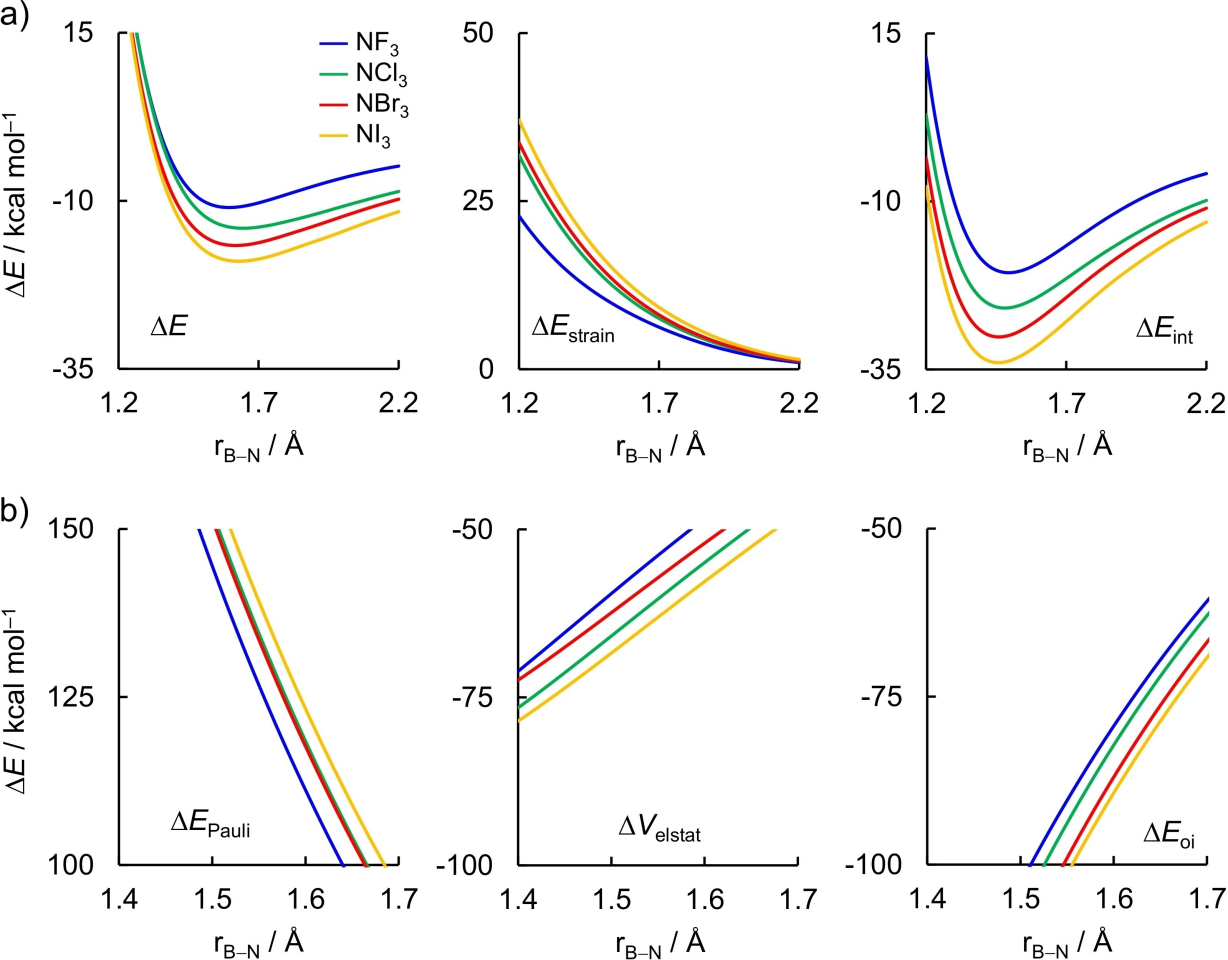
a) Activation strain model and b) energy decomposition analysis of the H_3_B−NY_3_ Lewis pairs projected onto the B−N bond distance (where Y = F, Cl, Br, and I) computed at ZORA‐BLYP‐D3(BJ)/TZ2P. Dispersion energy Δ*E*_disp_ not shown, see Table 4 for data at consistent geometries.

Finally, we turn our attention to the formation of Lewis adducts between borane and nitrogen trihalides. The activation strain model and energy decomposition analysis diagrams for the H_3_B−NY_3_ Lewis pairs (where Y = F, Cl, Br, and I) are shown in Figure 6, whereas data at consistent geometries is summarized in Table 4. The NI_3_ forms the strongest complex with borane and the energy of formation of the Lewis adduct Δ*E* decreases in strength, *i. e*., becomes less stabilizing, along the series: NI_3_, NBr_3_, NCl_3_, NF_3_. Trends in Δ*E* curves originate solely from a more stabilizing interaction energy Δ*E*_int_. Note that the strain energy Δ*E*_strain_ curves show a reversed trend, overruled by the trend in Δ*E*_int_, namely, NF_3_ has a less destabilizing Δ*E*_strain_ than NI_3_. Therefore, similar to borane−ammonia, the relative stability of the H_3_B−NY_3_ Lewis pairs is determined by Δ*E*_int_.

**Table 4:** Activation strain model and energy decomposition analysis terms (in kcal mol^−1^) computed at consistent geometries with a forming B−N distance of 1.687 Å of the H_3_B−NY_3_ Lewis pairs (where Y = F, Cl, Br, and I).^[a]^

Lewis base	Δ*E*	Δ*E*_strain_	Δ*E*_int_	Δ*V*_elstat_	Δ*E*_Pauli_	Δ*E*_oi_	Δ*E*_disp_
NF_3_	−10.5	6.5	−16.9	−39.6	88.2	−63.0	−2.5
NCl_3_	−14.0	7.8	−21.8	−46.0	94.6	−65.2	−5.2
NBr_3_	−16.3	8.4	−24.7	−43.6	94.2	−69.2	−6.1
NI_3_	−18.8	9.6	−28.3	−48.9	99.4	−71.5	−7.3

[a] Computed at ZORA‐BLYP‐D3(BJ)/TZ2P.

The observed trend in Δ*E*_int_ curves is given by the orbital interaction Δ*E*_oi_ curves, that is most stabilizing for the Lewis complex with NI_3_ and decreases in strength along the series NI_3_, NBr_3_, NCl_3_, NF_3_. From NI_3_ to NF_3_, at the consistent geometry (see Table 4), Δ*E*_int_ varies from a value of −28.3 to −16.9 kcal mol^−1^ and Δ*E*_oi_ varies from a value of −71.5 to −63.0 kcal mol^−1^. This is paralleled by a decrease of Pauli repulsion, that varies from a value of 99.4 to 88.2 kcal mol^−1^ from NI_3_ to NF_3_, as reflected by the decreasing number of core electrons and diffuse orbitals as the halogen decreases in size. Trends in Δ*V*_elstat_, on the other hand, are not exactly systematic along the Lewis bases. They are partially inverted and decreases in strength along the series NI_3_, NCl_3_, NBr_3_, NF_3_. Finally, the dispersion energy Δ*E*_disp_ has the smallest contribution to Δ*E*_int_ (not shown in Figure 6, see Table 4 for data at consistent geometries).

**Figure 7:**
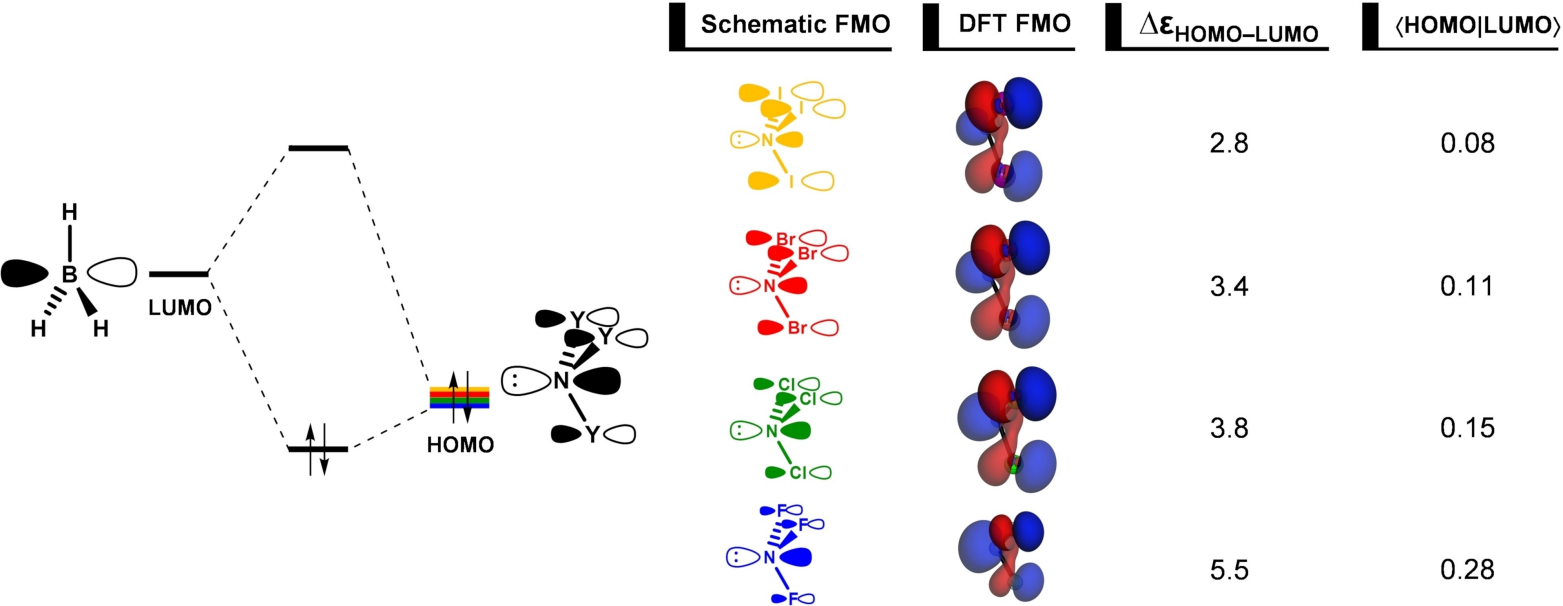
Schematic representation of the HOMO−LUMO orbital interaction in the H_3_B−NY_3_ Lewis pairs (where Y = F, Cl, Br, and I). Isosurface (at 0.03 au), energy gap (in eV) and orbital overlap of the interaction between HOMO and LUMO of the a_1_ irreducible representation of the *C*_3*v*_ symmetry.

Formation of the H_3_B−NY_3_ Lewis pairs involves a key orbital interaction between the filled out‐of‐phase mixing of N 2p_z_ and halogens *n*p_z_ orbitals of NY_3_ with the empty B 2p_z_ orbital of BH_3_, the HOMO(base)−LUMO(acid) interaction (see Figure 7; additional stabilizing contribution from the HOMO‐2(base)−LUMO(acid) interaction is given in Figure S4). However, this interaction is relatively less stabilizing compared to borane−ammonia. As the Y ligands vary from H to the increasingly more electronegative atoms I, Br, Cl and F, the HOMO drops in energy, which leads to a larger HOMO−LUMO energy gap (Δϵ_HOMO−LUMO_ = 2.5, 2.8, 3.4, 3.8, and 5.5 eV along NY_3_ = NH_3_, NI_3_, NBr_3_, NCl_3_, and NF_3_, respectively). The corresponding orbital overlap ⟨HOMO|LUMO⟩, on the other hand, decreases on descending group 17, *i. e*., it becomes less favorable. Because of the out‐of‐phase mixing of the *n*p_z_ orbitals, the amplitude of HOMO is larger on the less electronegative atom (either N or Y). Thus, the amplitude on the nitrogen atom decreases along the series NF_3_, NCl_3_, NBr_3_, and NI_3_, which decreases the spatial overlap with the empty 2p_z_ orbital of BH_3_. Therefore, the trend in Δϵ_HOMO−LUMO_ overrules the trend in ⟨HOMO|LUMO⟩, determining the trend in orbital interaction energies and, eventually, in the stability of the H_3_B−NY_3_ Lewis pairs.

## Conclusions

At variance with the current view, the strength of archetypical X_3_B−NY_3_ Lewis pair (where X,Y = H, F, Cl, Br, and I) bonds is not solely attributed to the strength of the stabilizing frontier molecular orbital interactions. The bonding mechanism involving boron trihalides, for example, is determined by the amount of energy required to deform the fragments, especially the Lewis acid, upon complexation. This follows from our detailed bonding analyses based on relativistic dispersion‐corrected density functional theory at ZORA‐BLYP‐D3(BJ)/TZ2P.

Our activation strain and quantitative Kohn−Sham MO analyses reveal that the bonding energy of the series X_3_B−NH_3_ is determined by the strain energy associated with the geometrical distortion of the Lewis acid on going from the planar to the pyramidal geometry acquired in the Lewis complex. We have, for the first time, quantitatively decomposed the strain energy of the Lewis acid in terms of the change in the interaction energy within one fragment upon its deformation. The decrease in the strain energy directly correlates with the weakening of the B−X bond as the electronegativity of X decreases along the series: F, Cl, Br, and I. Most of this effect arises from the bonding in the plane of the molecule, not in the π system as is widely believed. In other words, the less destabilizing energy required to deform a weak B−X bond results in a smaller strain energy, which manifests in a more stable Lewis pair. This is the actual reason why the Lewis pairs becomes systematically stronger as BX_3_ goes from BF_3_ to BI_3_, and not because of a more stabilizing interaction energy as is the currently accepted rationale. For the H_3_B−NY_3_ series, the bonding is driven by the charge‐transfer stemming from the commonly accepted HOMO−LUMO interaction between the lone pair on the nitrogen of the Lewis base and the empty p orbital at the boron of the Lewis acid.

This work clearly demonstrates the role of the strain energy, besides the well‐known donor−acceptor orbital and also electrostatic interactions, in playing a leading role in determining the strength of Lewis acid/base interactions. Our findings are both chemically intuitive and grounded in quantum chemical findings based on state‐of‐the‐art computations. Importantly, we have brought our understanding of these fundamental interactions into the 21^st^ century and hope that this work will be useful for the development of novel Lewis pair chemistries.

## Conflict of interest

The authors declare no conflict of interest.

## Supporting information

As a service to our authors and readers, this journal provides supporting information supplied by the authors. Such materials are peer reviewed and may be re‐organized for online delivery, but are not copy‐edited or typeset. Technical support issues arising from supporting information (other than missing files) should be addressed to the authors.

Supplementary

## References

[1a] T. L. Brown , H. E. LeMay , B. E. Bursten , J. R. Burdge , Chemistry: The Central Science, Pearson Prentice Hall, 2005;

[1b] P. Atkins , L. Jones , L. Laverman , Chemical Principles: The Quest for Insight, W. H. Freeman and Company, New York, 2016.

[2a] G. N. Lewis , Valence and the Structure of Atoms and Molecules, The Chemical Catalog Company, New York, 1923;

[2b] G. N. Lewis , J. Franklin Inst. 1938, 226, 293.

[3] W. B. Jensen , Chem. Rev. 1978, 78, 1.

[4] See, for instance:

[4a] P. Vermeeren , T. A. Hamlin , I. Fernández , F. M. Bickelhaupt , Angew. Chem. Int. Ed. 2020, 59, 6201; 10.1002/anie.201914582 PMC7187354 31944503

[4b] P. H. Huy , Eur. J. Org. Chem. 2020, 2020, 10;

[4c] T. A. Hamlin , I. Fernandez , F. M. Bickelhaupt , Angew. Chem. Int. Ed. 2019, 58, 8922; 10.1002/anie.201903196 PMC6617756 31033118

[4d] Q. Shi , W. Wang , Y. Wang , Y. Lan , C. Yao , D. Wei , Org. Chem. Front. 2019, 6, 2692.

[5] For examples on the applicability of frustrated Lewis pair catalysts, see:

[5a] J. Paradies , Coord. Chem. Rev. 2019, 380, 170;

[5b] D. W. Stephan , Science 2016, 354, 1248;

[5c] D. W. Stephan , J. Am. Chem. Soc. 2015, 137, 10018; 26214241 10.1021/jacs.5b06794

[5d] D. W. Stephan , G. Erker , Angew. Chem. Int. Ed. 2015, 54, 6400; 10.1002/anie.201409800 25974714

[5e] C. Appelt , H. Westenberg , F. Bertini , A. W. Ehlers , J. C. Slootweg , K. Lammertsma , W. Uhl , Angew. Chem. Int. Ed. 2011, 50, 3925; 10.1002/anie.201006901 21425419

[5f] D. W. Stephan , G. Erker , Angew. Chem. Int. Ed. 2010, 49, 46; 10.1002/anie.200903708 20025001

[5g] S. Grimme , H. Kruse , L. Goerigk , G. Erker , Angew. Chem. Int. Ed. 2010, 49, 1402; 10.1002/anie.200905484 20091722

[6a] M. Méndez , A. Cedillo , Comput. Theor. Chem. 2013, 1011, 44;

[6b] H. Anane , S. El Houssame , A. El Guerraze , A. Guermoune , A. Boutalib , A. Jarid , I. Nebot-Gil , F. Tomás , Cent. Eur. J. Chem. 2008, 6, 400;

[6c] H. Anane , S. El Houssame , A. El Guerraze , A. Jarid , A. Boutalib , I. Nebot-Gil , F. Tomás , J. Mol. Struct. 2004, 709, 103;

[6d] Y. Mo , L. Song , W. Wu , Q. Zhang , J. Am. Chem. Soc. 2004, 126, 3974; 15038752 10.1021/ja039778l

[6e] Y. Mo , J. Gao , J. Phys. Chem. A 2001, 105, 6530;

[6f] H. Hirao , H. Fujimoto , J. Phys. Chem. A 2000, 104, 6649;

[6g] S. Fau , G. Frenking , Mol. Phys. 1999, 96, 519;

[6h] D. J. Hankinson , J. Almlöf , K. R. Leopold , J. Phys. Chem. 1996, 100, 6904;

[6i] V. Jonas , G. Frenking , M. T. Reetz , J. Am. Chem. Soc. 1994, 116, 8141, and references cited therein;

[6j] G. Frenking , K. Wichmann , N. Fröhlich , C. Loschen , M. Lein , J. Frunzke , V. M. Rayón , Coord. Chem. Rev. 2003, 238–239, 5.

[7a] R. G. Pearson , J. Chem. Educ. 1968, 45, 643;

[7b] R. G. Pearson , Science 1966, 151, 172;17746330

[7c] R. G. Pearson , J. Am. Chem. Soc. 1963, 85, 3533.

[8a] T. Bettens , M. Alonso , F. De Proft , T. A. Hamlin , F. M. Bickelhaupt , Chem. Eur. J. 2020, 26, 3884; 31957943 10.1002/chem.202000272 PMC7154642

[8b] H. Mayr , M. Breugst , A. R. Ofial , Angew. Chem. Int. Ed. 2011, 50, 6470; 10.1002/anie.201007100 21726020

[8c] M. Breugst , H. Zipse , J. P. Guthrie , H. Mayr , Angew. Chem. Int. Ed. 2010, 49, 5165; 10.1002/anie.201001574 20568073

[9a] S. J. Grabowski , J. Comput. Chem. 2018, 39, 472; 28857264 10.1002/jcc.25056

[9b] B. J. van der Veken , E. J. Sluyts , J. Am. Chem. Soc. 1997, 119, 11516;

[9c] F. A. Cotton, G. Wilkinson, C. A. Murillo, M. Bochmann, *Advanced Inorganic Chemistry*, 6th ed., John Wiley and Sons, New York, **1999**.

[10a] A. Skancke , P. N. Skancke , J. Phys. Chem. 1996, 100, 15079;

[10b] V. Branchadell , A. Oliva , J. Mol. Struct. 1991, 236, 75;

[10c] J. F. Liebman , Struct. Chem. 1990, 1, 395.

[11a] J. A. Plumley , J. D. Evanseck , J. Phys. Chem. A 2009, 113, 5985; 19388700 10.1021/jp811202c

[11b] F. Bessac , G. Frenking , Inorg. Chem. 2003, 42, 7990; 14632517 10.1021/ic034141o

[11c] H. Hirao , K. Omoto , H. Fujimoto , J. Phys. Chem. A 1999, 103, 5807;

[11d] T. Brinck , J. S. Murray , P. Politzer , Inorg. Chem. 1993, 32, 2622;

[11e] I. Alkorta , J. Elguero , J. E. Del Bene , O. Mó , M. Yáñez , Chem. Eur. J. 2010, 16, 11897. 20836095 10.1002/chem.201001254

[12a] E. A. Robinson , G. L. Heard , R. J. Gillespie , J. Mol. Struct. 1999, 485–486, 305;

[12b] B. D. Rowsell , R. J. Gillespie , G. L. Heard , Inorg. Chem. 1999, 38, 4659. 11671188 10.1021/ic990713m

[13a] A. R. Jupp , T. C. Johnstone , D. W. Stephan , Inorg. Chem. 2018, 57, 14764; 30422644 10.1021/acs.inorgchem.8b02517

[13b] S. Noorizadeh , E. Shakerzadeh , J. Mol. Struct. 2008, 868, 22.

[14] G. Frenking , S. Fau , C. M. Marchand , H. Grützmacher , J. Am. Chem. Soc. 1997, 119, 6648.

[15] S. C. C. van der Lubbe , C. Fonseca Guerra , Chem. Asian J. 2019, 14, 2760. 31241855 10.1002/asia.201900717 PMC6771679

[16a] G. te Velde , F. M. Bickelhaupt , E. J. Baerends , C. Fonseca Guerra , S. J. A. van Gisbergen , J. G. Snijders , T. Ziegler , J. Comput. Chem. 2001, 22, 931;

[16b] C. Fonseca Guerra , J. G. Snijders , G. te Velde , E. J. Baerends , Theor. Chem. Acc. 1998, 99, 391;

[16c] ADF2017.103, SCM Theoretical Chemistry, Vrije Universiteit: Amsterdam (Netherlands).

[17a] A. D. Becke , Phys. Rev. A 1988, 38, 3098; 10.1103/physreva.38.3098 9900728

[17b] C. T. Lee , W. T. Yang , R. G. Parr , Phys. Rev. B 1988, 37, 785. 10.1103/physrevb.37.785 9944570

[18a] S. Grimme , J. Antony , S. Ehrlich , H. Krieg , J. Chem. Phys. 2010, 132, 154104; 20423165 10.1063/1.3382344

[18b] S. Grimme , S. Ehrlich , L. Goerigk , J. Comput. Chem. 2011, 32, 1456. 21370243 10.1002/jcc.21759

[19] E. R. Johnson , A. D. Becke , J. Chem. Phys. 2005, 123, 024101. 10.1063/1.1949201 16050735

[20a] E. van Lenthe , R. van Leeuwen , E. J. Baerends , J. G. Snijders , Int. J. Quantum Chem. 1996, 57, 281;

[20b] E. van Lenthe , E. J. Baerends , J. G. Snijders , J. Chem. Phys. 1994, 101, 9783.

[21] E. van Lenthe , E. J. Baerends , J. Comput. Chem. 2003, 24, 1142. 12759913 10.1002/jcc.10255

[22] M. Franchini , P. H. T. Philipsen , E. van Lenthe , L. Visscher , J. Chem. Theory Comput. 2014, 10, 1994. 26580526 10.1021/ct500172n

[23] M. Franchini , P. H. T. Philipsen , L. Visscher , J. Comput. Chem. 2013, 34, 1819. 23720371 10.1002/jcc.23323

[24a] A. Bérces , R. M. Dickson , L. Fan , H. Jacobsen , D. Swerhone , T. Ziegler , Comput. Phys. Commun. 1997, 100, 247;

[24b] H. Jacobsen , A. Bérces , D. P. Swerhone , T. Ziegler , Comput. Phys. Commun. 1997, 100, 263;

[24c] S. K. Wolff , Int. J. Quantum Chem. 2005, 104, 645.

[25] C. Y. Legault, CYLview 1.0b, Université de Sherbrooke: Sherbrooke, **2009**.

[26a] P. Vermeeren , S. C. C. van der Lubbe , C. Fonseca Guerra , F. M. Bickelhaupt , T. A. Hamlin , Nat. Protoc. 2020, 15, 649; 31925400 10.1038/s41596-019-0265-0

[26b] F. M. Bickelhaupt , K. N. Houk , Angew. Chem. Int. Ed. 2017, 56, 10070; 10.1002/anie.201701486 PMC5601271 28447369

[26c] L. P. Wolters , F. M. Bickelhaupt , WIREs Comput. Mol. Sci. 2015, 5, 324; 10.1002/wcms.1221 PMC4696410 26753009

[26d] I. Fernández , F. M. Bickelhaupt , Chem. Soc. Rev. 2014, 43, 4953; 24699791 10.1039/c4cs00055b

[26e] W.-J. van Zeist , F. M. Bickelhaupt , Org. Biomol. Chem. 2010, 8, 3118; 20490400 10.1039/b926828f

[26f] F. M. Bickelhaupt , J. Comput. Chem. 1999, 20, 114.

[27a] R. van Meer , O. V. Gritsenko , E. J. Baerends , J. Chem. Theory Comput. 2014, 10, 4432; 26588140 10.1021/ct500727c

[27b] F. M. Bickelhaupt , E. J. Baerends , in Rev. Comput. Chem., ed. K. B. Lipkowitz, D. B. Boyd, Wiley, Hoboken, 2000, pp. 1–86;

[27c] L. Zhao , M. von Hopffgarten , D. M. Andrada , G. Frenking , WIREs Comput. Mol. Sci. 2018, 8, e1345.

[28a] W.-J. van Zeist , C. Fonseca Guerra , F. M. Bickelhaupt , J. Comb. Chem. 2008, 29, 312; 10.1002/jcc.20786 17557284

[28b] X. Sun , T. M. Soini , J. Poater , T. A. Hamlin , F. M. Bickelhaupt , J. Comb. Chem. 2019, 40, 2227. 10.1002/jcc.25871 PMC6771738 31165500

[29] C. Fonseca Guerra , J.-W. Handgraaf , E. J. Baerends , F. M. Bickelhaupt , J. Comput. Chem. 2004, 25, 189. 14648618 10.1002/jcc.10351

[30] L. R. Thorne , R. D. Suenram , F. J. Lovas , J. Chem. Phys. 1983, 78, 167.

[31] For a detailed discussion on the behavior of each EDA term along formation of a chemical bond, see:

[31a] G. Frenking , F. M. Bickelhaupt , in The Chemical Bond: Fundamental Aspects of Chemical Bonding, ed. G. Frenking, S. Shaik, Wiley-VCH, Weinheim, 2014, 121–158;

[31b] A. Krapp , F. M. Bickelhaupt , G. Frenking , Chem. Eur. J. 2006, 12, 9196. 17024702 10.1002/chem.200600564

[32] T. A. Albright , J. K. Burdett , W. H. Wangbo , Orbital Interactions in Chemistry, Wiley, New York, 2013.

[33] V. Branchadell , A. Sbai , A. Oliva , J. Phys. Chem. 1995, 99, 6472.

[34] F. M. Bickelhaupt , T. Ziegler , P. von Ragué Schleyer , Organometallics 1996, 15, 1477.

[35] W.-J. van Zeist , R. Yi , F. M. Bickelhaupt , Sci. China Chem. 2010, 53, 210.

[36a] L. Pauling , The Nature of the Chemical Bond, Cornell University Press, Ithaca, NY, 1960;

[36b] A. L. Allred , J. Inorg. Nucl. Chem. 1961, 17, 215.

[37] Y. R. Luo , Comprehensive Handbook of Chemical Bond Energies, CRC Press, Boca Raton, FL, 2007.

