# Supplementary material for: Nature and Strength of Lewis Acid/Base Interaction in Boron and Nitrogen Trihalides
Source: Chem Asian J. 2020 Oct 21;15(23):4043–54. doi: 10.1002/asia.202001127 (PMC7756781; doi:10.1002/asia.202001127)
Supplement: Supplementary file 1 — Supplementary [file ASIA-15-4043-s001.pdf]

CHEMISTRY
AN ASIAN JOURNAL

Supporting Information

Nature and Strength of Lewis Acid/Base Interaction in Boron and Nitrogen Trihalides

Daniela Rodrigues Silva[+], Lucas de Azevedo Santos[+], Matheus P. Freitas, Célia Fonseca Guerra, and Trevor A. Hamlin*This manuscript is part of a special collection dedicated to Early Career Researchers.© 2020 The Authors. Chemistry - An Asian Journal published by Wiley-VCH GmbH. This is an open access article under the terms of the Creative Commons Attribution License, which permits use, distribution and reproduction in any medium, provided the original work is properly cited.

Contents

Supporting methods

Figure S1. Three-layer fragmentation scheme for activation strain and energy decomposition analyses. Inside each layer, the fragment analysis is done up to three fragments: Fragment 1 (blue), Fragment 2 (red) and Fragment 3 (green).

Table S1. Bond lengths (in Å), angles (in degrees) and energies (in kcal mol^{-1}) of the $X_3B–NY_3$ Lewis pairs (where X,Y = H, F, Cl, Br, and I) relative to the separated Lewis acids and Lewis bases.

Table S2. Energy decomposition analysis terms (in kcal mol^{-1}) computed at consistent geometries with a B–N distance of 1.687 Å of the $X_3B–NH_3$ Lewis pairs (where X = F, Cl, Br, and I).

Figure S2. Step-by-step deformation of the Lewis acid from the planar to the pyramidal geometry. Computed at ZORA-BLYP-D3(BJ)/TZ2P.

Table S3. The strain energy terms (in kcal mol^{-1}) associated with the step-by-step deformation of the Lewis acid from the planar to the pyramidal geometry and corresponding change in X–X distance (in Å).

Table S4. Energies (in kcal mol^{-1}), orbital energy gap (in eV) and orbital overlap of the analysis of the bonding mechanism in planar and pyramidal BX_3 (where X = F, Cl, Br, and I).

Figure S3. Orbital interaction scheme for planar and pyramidal BX_3 (where X = F, Cl, Br, and I).

Figure S4. Schematic representation of the HOMO-2(base)–LUMO(acid) orbital interaction in the $H_3B–NY_3$ Lewis pairs (where Y = F, Cl, Br, and I). Isosurface (at 0.03 au), energy gap (in eV) and orbital overlap of the interaction between HOMO-2 and LUMO of the a_1 irreducible representation of the C_{3v} symmetry.

Table S5. Cartesian coordinates (Å), energies (kcal mol^{-1}), and the number of imaginary vibrational frequencies of the optimized Lewis acid, Lewis bases and $X_3B–NY_3$ Lewis pairs (where X,Y = H, F, Cl, Br, and I) computed at ZORA-BLYP-D3(BJ)/TZ2P.

Supporting methods

Thermochemistry

Bond enthalpies at 298.15 K and 1 atm ($\Delta H_{298.15}$) were calculated from electronic bond energies (ΔE) and vibrational frequencies using standard thermochemistry relations for an ideal gas [Eq. (S1)].[1]

$$\Delta H_{298.15} = \Delta E + \Delta E_{\mathrm{trans},298.15} + \Delta E_{\mathrm{rot},298.15} + \Delta E_{\mathrm{vib},0} + \Delta(\Delta E_{\mathrm{vib},0})_{298.15} + \Delta(pV) \quad \text{(S1)}$$

Here, $\Delta E_{\mathrm{trans},298.15}$, $\Delta E_{\mathrm{rot},298.15}$, and $\Delta E_{\mathrm{vib},0}$ are the differences between the Lewis pair minus the isolated Lewis acid and base, that results from forming the B–N bond, in translational, rotational, and zero-point vibrational energy, respectively. The last term, $\Delta(\Delta E_{\mathrm{vib},0})_{298.15}$, is the change in the vibrational energy difference when going from 0 K to 298.15 K. The vibrational energy corrections are based on our frequency calculations. The molar work term $\Delta(pV)$ is $(\Delta n)RT$; $\Delta n = -1$ for two fragments BX_3 and NY_3 combining to one molecule $X_3B–NY_3$. Thermal corrections for the electronic energy are neglected.

Decomposition of the strain energy

The decomposition of the strain energy provides quantum chemical insights to the structural deformation of the molecular fragments along the activation strain analysis of a specific interaction or reaction. In the formation of the $X_3B–NH_3$ Lewis pairs (where X = F, Cl, Br, and I), we schematically divide the decomposition analysis of the BX_3 strain energy $\Delta E_{\mathrm{strain},BX_3}$ into three Layers, in which the Layer 1 corresponds to the bonding of BX_3 with NH_3, the Layer 2 corresponds to the bonding between B and X_3, and the Layer 3 corresponds to the interaction between the three X atoms (Figure S1). Using this multi-layer fragment analysis, we show that the strain of the BX_3 molecule in Layer 1 ($\Delta E_{\mathrm{strain},BX_3}$) is written in terms of the change in the interaction energy between B and X_3 in Layer 2 ($\Delta\Delta E_{\mathrm{int},B–X_3}$) and the change in the interaction energy between the X atoms in Layer 3 ($\Delta\Delta E_{\mathrm{int},X–X–X}$) when BX_3 goes from planar to pyramidal geometry [see Eq. (S2) in this Supporting Information or Table 3 in the main text].

$$\Delta E_{\mathrm{strain},BX_3} = \Delta\Delta E_{\mathrm{int},B–X_3} + \Delta\Delta E_{\mathrm{int},X–X–X} \quad \text{(S2)}$$

1 a) P. W. Atkins, J. de Pauli, *Physical Chemistry*, Edn. 9, Oxford University Press, Oxford, **2010**; b) F. Jensen, *Introduction to Computational Chemistry*, Edn. 2, Wiley, West Sussex, **2007**.

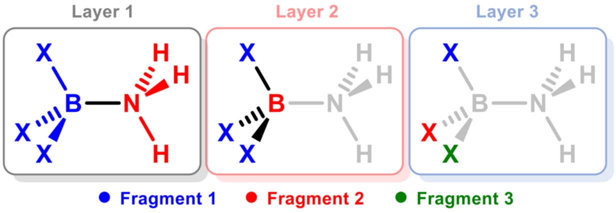

Figure S1. Three-layer fragmentation scheme for activation strain and energy decomposition analyses. Inside each layer, the fragment analysis is done up to three fragments: Fragment 1 (blue), Fragment 2 (red) and Fragment 3 (green).

Layer 1

In Layer 1, we have the bonding between BX_3 and NH_3 to form the $X_3B–NH_3$ Lewis pair (Figure S1). Here, the analysis is done by dividing the complex into two fragments: Fragment 1, in blue, is the BX_3 molecule and Fragment 2, in red, is the NH_3 molecule. The total bonding energy in Layer 1 ΔE_{Layer1} is, therefore, equivalent to the bonding energy between BX_3 and NH_3 $\Delta E_{X_3B–NH_3}$. Ultimately, $\Delta E_{X_3B–NH_3}$ is decomposed according to the activation strain model scheme [Eq. (S3.1)].

$$\Delta E_{\text{Layer1}} = \Delta E_{X_3B–NH_3} = \Delta E_{\text{strain},X_3B–NH_3} + \Delta E_{\text{int},X_3B–NH_3} \tag{S3.1}$$

The strain energy ΔE_{strain} is defined as the difference between the sum of the energies of each fragment in the geometry of the complex and the sum of the energies of each fragment in its own equilibrium geometry. Then, the strain energy for the complex $X_3B–NH_3$ $\Delta E_{\text{strain},X_3B–NH_3}$ is written as:

$$\Delta E_{\text{strain},X_3B–NH_3} = [E_{BX_3}(\text{pyram}) + E_{NH_3}(\text{pyram})] – [E_{BX_3}(\text{planar}) + E_{NH_3}(\text{pyram*})] \tag{S3.2}$$

Here, we write their geometry in the complex as E(pyram), since they adopt a pyramidal geometry in the complex. At the equilibrium geometry, the energy of the BX_3 fragment is written as E_{BX_3}(planar), since it adopts a planar geometry, and the energy of the NH_3 fragment, which is already pyramidal, is written as E_{NH_3}(pyram*). Rearranging the Eq. (S3.2), note that $\Delta E_{\text{strain},X_3B–NH_3}$ can also be written as the sum of the strain energy of each fragment when both go from their equilibrium geometry to the geometry they adopt in the complex [Eq. (S3.4)].

$$\Delta E_{\text{strain},X_3B–NH_3} = [E_{BX_3}(\text{pyram}) – E_{BX_3}(\text{planar})] + [E_{NH_3}(\text{pyram}) – E_{NH_3}(\text{pyram*})] \tag{S3.3}$$

$$\Delta E_{\text{strain,X}_3\text{B–NH}_3} = \Delta E_{\text{strain,BX}_3} + \Delta E_{\text{strain,NH}_3} \quad \text{(S3.4)}$$

The strain energy of the BX_3 fragment is then defined as:

$$\Delta E_{\text{strain,BX}_3} = E_{\text{BX}_3}(\text{pyram}) - E_{\text{BX}_3}(\text{planar}) \quad \text{(S3.5)}$$

$\Delta E_{\text{strain,BX}_3}$ is the quantity we want to quantum chemically understand.

Layer 2

The next step is to scale down to Layer 2, where we have the BX_3 molecule, and not the whole X_3B–NH_3 complex, taken as our system. Here, the analysis is done dividing the BX_3 molecule into two open-shell fragments: Fragment 1, in blue, is the $(X\bullet)_3$ molecule in its quartet valence configuration and in the geometry which it acquires in the overall molecule and Fragment 2, in red, is the B-sp^2 atom (see Figure S1). In other words, we analyze here the interaction between B-sp^2 and $(X\bullet)_3$. In our analysis, we recall that the BX_3 molecule exists in two different geometries: planar, the one in its equilibrium geometry, and pyramidal, the one it adopts in the complex. Thus, there will be a total bonding energy in Layer 2 for each planar and pyramidal geometries written as $\Delta E_{\text{Layer2}}(\text{planar})$ and $\Delta E_{\text{Layer2}}(\text{pyram})$, respectively. Note that there is no geometrical deformation inside Layer 2. Therefore, when proceeding with the activation strain analysis on each $\Delta E_{\text{Layer2}}(\text{planar})$ and $\Delta E_{\text{Layer2}}(\text{pyram})$ Layer 2 energies, their strain energy is zero and the total bonding energy is equivalent to the interaction energy [see Eq. (S4.1) and (S4.2)].

$$\Delta E_{\text{Layer2}}(\text{planar}) = \Delta E_{\text{int,B–X}_3}(\text{planar}) \quad \text{(S4.1)}$$

$$\Delta E_{\text{Layer2}}(\text{pyram}) = \Delta E_{\text{int,B–X}_3}(\text{pyram}) \quad \text{(S4.2)}$$

The interaction energy ΔE_{int} is defined as the difference between the energy of the system and the sum of the energies of the fragments in the geometry they adopt in the system. Thus, the interaction energy between B-sp^2 and $(X\bullet)_3$ in each planar and pyramidal geometry is written as, respectively:

$$\Delta E_{\text{int,B–X}_3}(\text{planar}) = E_{\text{BX}_3}(\text{planar}) - [E_{\text{B}}(\text{planar}) + E_{\text{X}_3}(\text{planar})] \quad \text{(S4.3)}$$

$$\Delta E_{\text{int,B–X}_3}(\text{pyram}) = E_{\text{BX}_3}(\text{pyram}) - [E_{\text{B}}(\text{pyram}) + E_{\text{X}_3}(\text{pyram})] \quad \text{(S4.4)}$$

Rearranging the Eq. (S4.3) and (S4.4) we have:

$$E_{\text{BX}_3}(\text{planar}) = \Delta E_{\text{int,B–X}_3}(\text{planar}) + E_{\text{B}}(\text{plan}) + E_{\text{X}_3}(\text{plan}) \quad \text{(S4.5)}$$

$$E_{\text{BX}_3}(\text{pyram}) = \Delta E_{\text{int,B–X}_3}(\text{pyram}) + E_{\text{B}}(\text{pyram}) + E_{\text{X}_3}(\text{pyram}) \quad \text{(S4.6)}$$

Here, we have expressions to define the energy of the BX_3 molecule in the planar E_{BX_3}(plan) and pyramidal E_{BX_3}(pyram) geometries. Note that the strain energy of the BX_3 molecule $\Delta E_{strain,BX_3}$ is defined in terms of E_{BX_3}(plan) and E_{BX_3}(pyram) [Eq. (S3.5)], which now can be rewritten as:

$$\Delta E_{\text{strain,BX}_3} = \Delta E_{\text{int,B–X}_3}(\text{pyram}) + E_{\text{B}}(\text{pyram}) + E_{\text{X}_3}(\text{pyram}) - \Delta E_{\text{int,B–X}_3}(\text{plan}) - E_{\text{B}}(\text{plan}) - E_{\text{X}_3}(\text{plan}) \quad \text{(S4.7)}$$

Rearranging the Eq. (S4.7) we have:

$$\Delta E_{\text{strain,BX}_3} = \Delta E_{\text{int,B–X}_3}(\text{pyram}) - \Delta E_{\text{int,B–X}_3}(\text{plan}) + E_{\text{B}}(\text{pyram}) - E_{\text{B}}(\text{plan}) + E_{\text{X}_3}(\text{pyram}) - E_{\text{X}_3}(\text{plan}) \quad \text{(S4.8)}$$

The difference between the two first terms in Eq. (S4.8), $\Delta E_{int,B–X_3}$(pyram) and $\Delta E_{int,B–X_3}$(plan), will give the change in the interaction energy between B-sp^2 and $(X\bullet)_3$ when the BX_3 molecule goes from planar to pyramidal geometry, namely, $\Delta\Delta E_{int,B–X_3}$. The difference between the terms E_B(pyram) and E_B(planar) can be understood as the strain energy of the atom B-sp^2 when it goes from planar to pyramidal geometry. Of course, an atom cannot undergo any structural deformation. As result, E_B(pyram) – E_B(planar) is zero. The difference between E_{X_3}(pyram) and E_{X_3}(planar) is the strain energy of the $(X\bullet)_3$ molecule when it goes from its geometry adopted in the planar BX_3 to the pyramidal BX_3, namely, $\Delta E_{strain,X_3}$. As result, $\Delta E_{strain,BX_3}$ is written as:

$$\Delta E_{\text{strain,BX}_3} = \Delta\Delta E_{\text{int,B–X}_3} + \Delta E_{\text{strain,X}_3} \quad \text{(S4.9)}$$

Now we have another strain energy term, $\Delta E_{strain,X_3}$, that needs a quantum chemical meaning, defined as:

$$\Delta E_{strain,X_3} = E_{X_3}(pyram) - E_{X_3}(planar) \quad (S4.10)$$

Layer 3

The next and last step is to scale down to Layer 3, where the $(X\bullet)_3$ molecule is taken as our system. Here, we analyze the interaction between the three X atoms dividing the $(X\bullet)_3$ molecule in its quartet valence configuration into three open-shell fragments: each Fragment 1, in blue, Fragment 2, in red, and Fragment 3, in green, is an X atom in its doublet valence configuration (see Figure S1). Again, the $(X\bullet)_3$ molecule exists in two different geometries: the one it adopts in the planar BX_3 and the one it adopts in the pyramidal BX_3. Analogously to the Layer 2, there will be a total bonding energy in Layer 3 for each geometry, $\Delta E_{Layer3}(planar)$ and $\Delta E_{Layer3}(pyram)$, that is equivalent to their respective interaction energy [Eq. (S5.1) and (S5.2)].

$$\Delta E_{Layer3}(plan) = \Delta E_{int,X-X-X}(planar) \quad (S5.1)$$

$$\Delta E_{Layer3}(pyram) = \Delta E_{int,X-X-X}(pyram) \quad (S5.2)$$

The interaction energy between the three X atoms in the $(X\bullet)_3$ molecule in each planar and pyramidal geometry is written as:

$$\Delta E_{int,X-X-X}(planar) = E_{X-X-X}(planar) - 3E_X \quad (S5.3)$$

$$\Delta E_{int,X-X-X}(pyram) = E_{X-X-X}(pyram) - 3E_X \quad (S5.4)$$

Rearranging the Eq. (S5.3) and (S5.4) we have:

$$E_{X_3}(planar) = \Delta E_{int,X-X-X}(planar) + 3E_X \quad (S5.5)$$

$$E_{X_3}(pyram) = \Delta E_{int,X-X-X}(pyram) + 3E_X \quad (S5.6)$$

Here, we have expressions to define the energy of the $(X\bullet)_3$ molecule its quartet valence configuration in each planar E_{X_3}(planar) and pyramidal E_{X_3}(pyram) geometries. Therefore, the strain energy of the $(X\bullet)_3$ molecule $\Delta E_{strain,X_3}$ [Eq. (S4.10)] can be written as:

$$\Delta E_{strain,X_3} = \Delta E_{int,X-X-X}(\text{pyram}) - \Delta E_{int,X-X-X}(\text{planar}) + 3E_X - 3E_X \quad \text{(S5.7)}$$

The difference between the two first terms in Eq. (S5.7), $\Delta E_{int,X-X-X}$(pyram) – $\Delta E_{int,X-X-X}$(planar), will give the change in the interaction energy between the three X atoms in their doublet valence configuration when the BX_3 molecule goes from planar to pyramidal geometry, namely, $\Delta\Delta E_{int,X-X-X}$. Notably, the difference between $3E_X$ – $3E_X$ is zero. As result, $\Delta E_{strain,X_3}$ is equal to $\Delta\Delta E_{int,X-X-X}$.

$$\Delta E_{strain,X_3} = \Delta\Delta E_{int,X-X-X} \quad \text{(S5.8)}$$

Finally, we use the Eq. (S5.8) to rewrite the Eq. (S4.9), yielding the Eq. (S2) (see also Table 3 in the main text), which gives the strain of the BX_3 molecule $\Delta E_{strain,BX_3}$ in terms of $\Delta\Delta E_{int,B-X_3}$ and $\Delta\Delta E_{int,X-X-X}$.

Table S1. Bond lengths (in Å), angles (in degrees) and energies (in kcal mol^{-1}) of the X_3B–NY_3 Lewis pairs (where X,Y = H, F, Cl, Br, and I) in their equilibrium geometry relative to the separated Lewis acids and Lewis bases.[a]

X_3B–NY_3	$r_{B–X}$ [b]	$\Delta r_{B–X}$ [c]	$\theta_{X–B–X}$ [b]	$\Delta\theta_{X–B–X}$ [c]	$r_{N–Y}$ [b]	$\Delta r_{N–Y}$ [c]	$\theta_{Y–N–Y}$ [b]	$\Delta\theta_{Y–N–Y}$ [c]	$r_{B–N}$	$\Delta H_{298.15}$	ΔE	ΔE_{strain}	ΔE_{int}
H_3B–NH_3	1.193	0.018	120.0	–6.2	1.022	0.000	106.7	1.1	1.675	–25.7	–29.5	12.5	–42.0
F_3B–NH_3	1.327	0.058	120.0	–5.5	1.022	0.001	106.7	2.0	1.718	–16.1	–18.4	21.1	–39.5
Cl_3B–NH_3	1.758	0.094	120.0	–6.3	1.022	0.003	106.7	2.2	1.642	–18.3	–21.2	21.7	–42.9
Br_3B–NH_3	1.922	0.102	120.0	–6.5	1.022	0.004	106.7	2.2	1.622	–22.0	–25.0	19.7	–44.7
I_3B–NH_3	2.145	0.110	120.0	–6.4	1.022	0.005	106.7	2.0	1.617	–23.2	–26.2	16.9	–43.2
H_3B–NF_3	1.193	0.010	120.0	–4.2	1.421	–0.008	101.7	0.6	1.595	–8.7	–11.0	8.3	–19.3
H_3B–NCl_3	1.193	0.012	120.0	–4.5	1.826	0.004	107.3	–0.9	1.645	–12.0	–14.1	8.9	–23.0
H_3B–NBr_3	1.193	0.015	120.0	–5.1	1.991	0.009	108.3	–1.6	1.618	–14.8	–16.7	10.4	–27.0
H_3B–NI_3	1.193	0.017	120.0	–5.4	2.183	0.014	110.5	–2.5	1.630	–17.2	–19.0	11.3	–30.4

[a] Computed at ZORA-BLYP-D3(BJ)/TZ2P. [b] Bond lengths and angles as in the optimized equilibrium geometry of the isolated BX_3 and NY_3. [c] Change in the geometrical parameters upon formation of the Lewis pair, relative to the isolated BX_3 and NY_3.

Table S2. Energy decomposition analysis terms (in kcal mol^{-1}) computed at consistent geometries with a B–N distance of 1.687 Å of the X_3B–NH_3 Lewis pairs (where X = F, Cl, Br, and I).[a]

Lewis acid	ΔE_{int}	ΔV_{elstat}	ΔE_{Pauli}	ΔE_{oi}	ΔE_{disp}
BF_3	–40.6	–94.5	133.6	–77.1	–2.6
BCl_3	–41.3	–107.8	173.8	–102.1	–5.2
BBr_3	–42.6	–110.2	183.4	–109.7	–6.1
BI_3	–41.2	–114.9	197.6	–116.7	–7.2

[a] Computed at ZORA-BLYP-D3(BJ)/TZ2P.

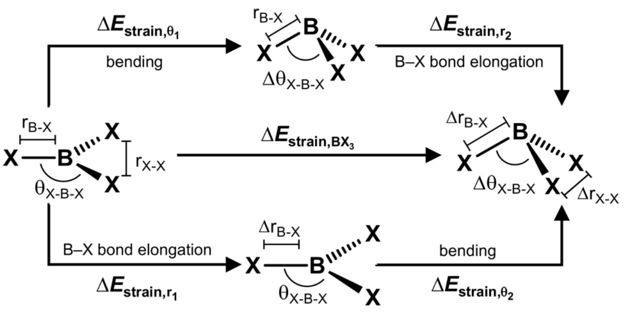

Figure S2. Step-by-step deformation of the Lewis acid from the planar to the pyramidal geometry. Computed at ZORA-BLYP-D3(BJ)/TZ2P.

Table S3. The strain energy terms (in kcal mol[−1]) associated with the step-by-step deformation of the Lewis acid from the planar to the pyramidal geometry[a] and corresponding change in X–X distance (in Å)[b].

Lewis acid	$\Delta E_{\text{strain},\theta_1}$	$\Delta E_{\text{strain},r_2}$	$\Delta E_{\text{strain},r_1}$	$\Delta E_{\text{strain},\theta_2}$	$\Delta E_{\text{strain},BX_3}$	$\Delta \mathbf{r}_{X\text{–}X}$
BF_3	19.1	3.1	5.5	16.7	22.2	0.032
BCl_3	16.7	3.4	6.2	13.9	20.1	0.053
BBr_3	14.9	2.9	5.6	12.2	17.8	0.056
BI_3	12.8	2.5	4.9	10.4	15.3	0.057

[a] Geometry adopted in the complex with a B–N distance of 1.687 Å of the X_3B–NH_3 Lewis pairs (where X = F, Cl, Br, and I). [b] Computed at ZORA-BLYP-D3(BJ)/TZ2P.

Table S4. Energies (in kcal mol[−1]), orbital energy gap (in eV) and orbital overlap of the analysis of the bonding mechanism in planar and pyramidal BX_3 (where X = F, Cl, Br, and I).[a]

	BF_3		BCl_3		BBr_3		BI_3	
	plan	pyr	plan	pyr	plan	pyr	plan	pyr
$\Delta E_{\text{int},X\text{–}X\text{–}X}$	37.1	32.3	52.0	45.5	48.4	42.7	46.7	41.6
$\Delta E_{\text{int},B\text{–}X_3}$	–640.1	–613.1	–475.3	–448.6	–420.6	–397.0	–367.2	–346.7
$\Delta E_{\text{Pauli},B\text{–}X_3}$	922.6	780.2	635.9	509.9	570.7	448.7	495.9	383.8
$\Delta V_{\text{elstat},B\text{–}X_3}$	–494.0	–419.2	–454.3	–358.5	–450.7	–348.2	–415.4	–316.0
$\Delta E_{\text{oi},B\text{–}X_3}$	–1068.0	–973.3	–654.2	–597.3	–537.3	–494.2	–443.7	–410.4
$\Delta E_{\text{oi},a_1}$	–305.3	–278.3	–180.8	–167.4	–147.7	–139.3	–126.8	–121.0
$\Delta E_{\text{oi},e_1}$	–759.7	–692.6	–472.2	–429.2	–388.8	–354.5	–316.5	–289.2
$\Delta E_{\text{oi},a_2}$	–2.9	–2.3	–1.1	–0.8	–0.7	–0.5	–0.4	–0.3
$\Delta E_{\text{disp},B\text{–}X_3}$	–0.8	–0.8	–2.7	–2.7	–3.3	–3.3	–4.1	–4.1
$\langle 1a_1 \vert 2s\rangle$	0.41	0.39	0.53	0.49	0.56	0.52	0.57	0.52
$\langle 1e_1 \vert 2p_x\rangle$	0.34	0.32	0.40	0.36	0.40	0.36	0.40	0.36
$\langle 2a_1 \vert 2p_z\rangle$	0.45	0.38	0.47	0.37	0.46	0.36	0.44	0.34
$\vert\Delta\varepsilon_{1a_1\text{–}2s}\vert$	2.7	2.6	0.2	0.3	1.0	1.1	1.9	2.0
$\vert\Delta\varepsilon_{1e_1\text{–}2p_x}\vert$	8.1	8.0	5.1	5.0	4.3	4.2	3.4	3.3
$\vert\Delta\varepsilon_{2a_1\text{–}2p_z}\vert$	7.0	6.9	4.5	4.5	3.8	3.7	3.0	3.0

[a] Computed at ZORA-BLYP-D3(BJ)/TZ2P.

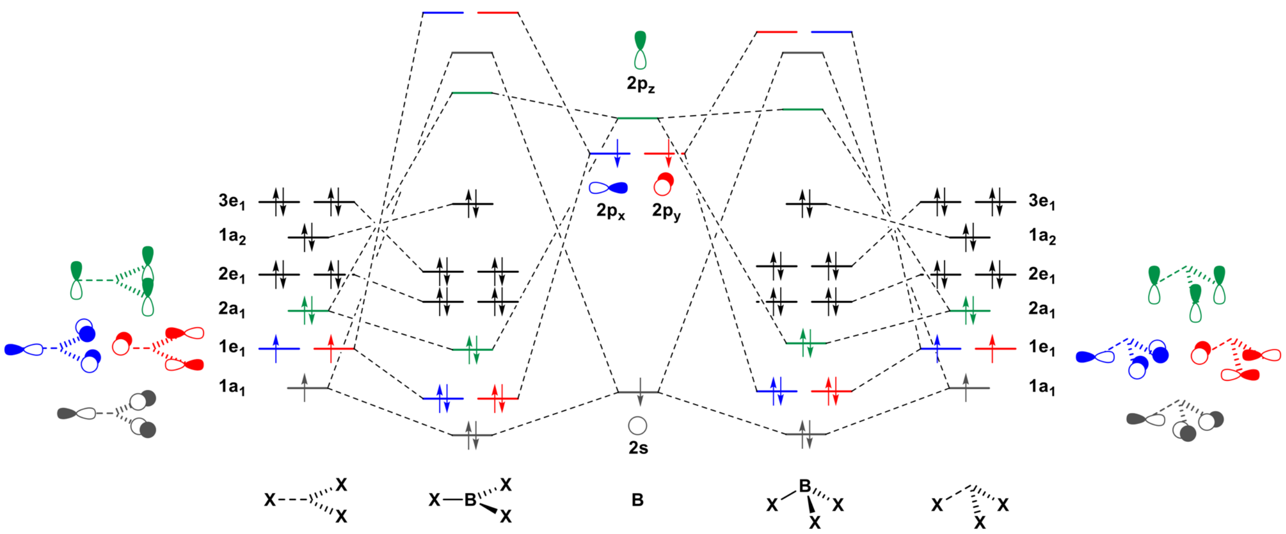

Figure S3. Orbital interaction scheme for planar and pyramidal BX_3 (where X = F, Cl, Br, and I).

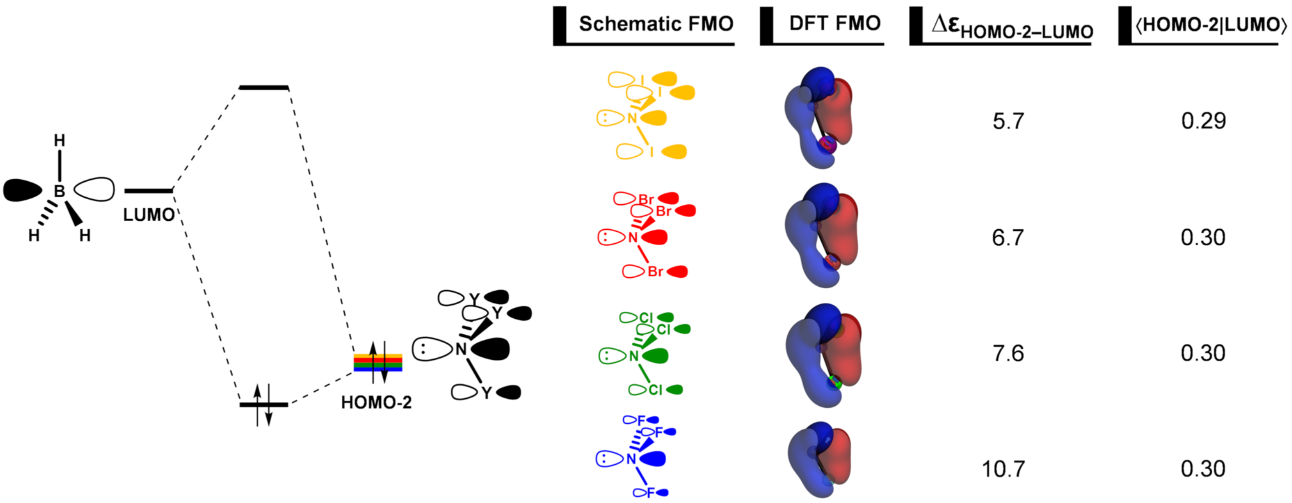

Figure S4. Schematic representation of the HOMO-2(base)–LUMO(acid) orbital interaction in the $H_3B–NY_3$ Lewis pairs (where Y = F, Cl, Br, and I). Isosurface (at 0.03 au), energy gap (in eV) and orbital overlap of the interaction between HOMO-2 and LUMO of the a_1 irreducible representation of the C_{3v} symmetry.

Table S5. Cartesian coordinates (Å), energies (kcal mol^{-1}), and the number of imaginary vibrational frequencies of the optimized Lewis acid, Lewis bases and X_3B–NY_3 Lewis pairs (where X,Y = H, F, Cl, Br, and I) computed at ZORA-BLYP-D3(BJ)/TZ2P.

BH_3
E = –358.12
H = –339.59
N_{imag} = 0

B	0.00000000	0.00000000	0.00000000
H	0.59641277	-1.03301722	0.00000000
H	0.59641277	1.03301722	0.00000000
H	-1.19282554	0.00000000	0.00000000

NH_3
E = –434.94
H = –411.73
N_{imag} = 0

N	0.00000000	0.00000000	0.35931838
H	0.94654541	0.00000000	-0.02597136
H	-0.47327270	-0.81973237	-0.02597136
H	-0.47327270	0.81973237	-0.02597136

H_3B–NH_3
E = –822.57
H = –777.02
N_{imag} = 0

N	0.00000000	0.00000000	0.74958555
H	-0.95392312	0.00000000	1.11822495
H	0.47696156	-0.82612165	1.11822495
H	0.47696156	0.82612165	1.11822495
B	0.00000000	0.00000000	-0.92533929
H	-0.58595881	-1.01491043	-1.23147301
H	-0.58595881	1.01491043	-1.23147301
H	1.17191762	0.00000000	-1.23147301

BF_3
E = –525.07
H = –514.83
N_{imag} = 0

B	0.00000000	0.00000000	0.00000000
F	0.66371319	-1.14958496	0.00000000
F	0.66371319	1.14958496	0.00000000
F	-1.32742637	0.00000000	0.00000000

F_3B–NH_3
E = –978.39
H = –942.64
N_{imag} = 0

N	0.00000000	0.00000000	0.80999140
H	-0.95957344	0.00000000	1.16408867
H	0.47978672	-0.83101498	1.16408867
H	0.47978672	0.83101498	1.16408867
B	0.00000000	0.00000000	-0.90795242
F	-0.67249809	-1.16480086	-1.23710174
F	-0.67249809	1.16480086	-1.23710174
F	1.34499618	0.00000000	-1.23710174

BCl_3
E = –346.03
H = –338.10
N_{imag} = 0

B	0.00000000	0.00000000	0.00000000
Cl	0.87894830	-1.52238311	0.00000000
Cl	0.87894830	1.52238311	0.00000000
Cl	-1.75789659	0.00000000	0.00000000

$Cl_3B–NH_3$
E = –802.18
H = –768.16
N_{imag} = 0

N	0.00000000	0.00000000	0.86629680
H	-0.96305435	0.00000000	1.21747079
H	0.48152718	-0.83402954	1.21747079
H	0.48152718	0.83402954	1.21747079
B	0.00000000	0.00000000	-0.77525098
Cl	-0.89492389	-1.55005365	-1.25027536
Cl	-0.89492389	1.55005365	-1.25027536
Cl	1.78984778	0.00000000	-1.25027536

BBr_3
E = –295.26
H = –287.96
N_{imag} = 0

B	0.00000000	0.00000000	0.00000000
Br	0.96111184	-1.66469455	0.00000000
Br	0.96111184	1.66469455	0.00000000
Br	-1.92222369	0.00000000	0.00000000

$Br_3B–NH_3$
E = –755.23
H = –721.68
N_{imag} = 0

N	0.00000000	0.00000000	0.91047440
H	-0.96381846	0.00000000	1.26238221
H	0.48190923	-0.83469127	1.26238221
H	0.48190923	0.83469127	1.26238221
B	0.00000000	0.00000000	-0.71147896
Br	-0.97747928	-1.69304378	-1.23713264
Br	-0.97747928	1.69304378	-1.23713264
Br	1.95495856	0.00000000	-1.23713264

BI_3
E = –243.61
H = –236.67
N_{imag} = 0

B	0.00000000	0.00000000	0.00000000
I	1.07259328	-1.85778606	0.00000000
I	1.07259328	1.85778606	0.00000000
I	-2.14518657	0.00000000	0.00000000

$I_3B–NH_3$
E = –704.78
H = –671.56
N_{imag} = 0

N	0.00000000	0.00000000	0.96455149

H	-0.96394071	0.00000000	1.31954197
H	0.48197036	-0.83479714	1.31954197
H	0.48197036	0.83479714	1.31954197
B	0.00000000	0.00000000	-0.65266033
I	-1.08931126	-1.88674244	-1.23314526
I	-1.08931126	1.88674244	-1.23314526
I	2.17862251	0.00000000	-1.23314526

NF_3
E = –344.00
H = –335.42
N_{imag} = 0

N	0.00000000	0.00000000	0.49950851
F	1.27275577	0.00000000	-0.13254697
F	-0.63637788	-1.10223883	-0.13254697
F	-0.63637788	1.10223883	-0.13254697

$H_3B–NF_3$
E = –713.13
H = –683.68
N_{imag} = 0

N	0.00000000	0.00000000	0.72424181
F	-0.63517836	1.10016120	1.34319542
F	-0.63517836	-1.10016120	1.34319542
F	1.27035673	0.00000000	1.34319542
B	0.00000000	0.00000000	-0.87059139
H	-1.17685488	0.00000000	-1.11992668
H	0.58842744	1.01918622	-1.11992668
H	0.58842744	-1.01918622	-1.11992668

NCl_3
E = –248.49
H = –241.51
N_{imag} = 0

N	0.00000000	0.00000000	0.44488066
Cl	1.69885141	0.00000000	-0.22522268
Cl	-0.84942571	-1.47124848	-0.22522268
Cl	-0.84942571	1.47124848	-0.22522268

$H_3B–NCl_3$
E = –620.71
H = –593.09
N_{imag} = 0

N	0.00000000	0.00000000	0.73923939
Cl	-0.84593437	1.46520130	1.43611161
Cl	-0.84593437	-1.46520130	1.43611161
Cl	1.69186873	0.00000000	1.43611161
B	0.00000000	0.00000000	-0.90540215
H	-1.17724224	0.00000000	-1.16356493
H	0.58862112	1.01952168	-1.16356493
H	0.58862112	-1.01952168	-1.16356493

NBr_3
E = –224.91
H = –218.24
N_{imag} = 0

N	0.00000000	0.00000000	0.43471953
Br	1.86323753	0.00000000	-0.26761955

Br	-0.93161876	-1.61361103	-0.26761955
Br	-0.93161876	1.61361103	-0.26761955

$H_3B–NBr_3$
E = –599.69
H = –572.60
N_{imag} = 0

N	0.00000000	0.00000000	0.73435888
Br	-0.92666533	1.60503144	1.48814554
Br	-0.92666533	-1.60503144	1.48814554
Br	1.85333066	0.00000000	1.48814554
B	0.00000000	0.00000000	-0.88371109
H	-1.17598019	0.00000000	-1.16025952
H	0.58799010	1.01842872	-1.16025952
H	0.58799010	-1.01842872	-1.16025952

NI_3
E = –205.07
H = –198.58
N_{imag} = 0

N	0.00000000	0.00000000	0.36380756
I	2.07134127	0.00000000	-0.32646524
I	-1.03567063	-1.79383416	-0.32646524
I	-1.03567063	1.79383416	-0.32646524

$H_3B–NI_3$
E = –582.19
H = –555.39
N_{imag} = 0

N	0.00000000	0.00000000	0.75631124
I	-1.02696740	1.77875971	1.53750182
I	-1.02696740	-1.77875971	1.53750182
I	2.05393479	0.00000000	1.53750182
B	0.00000000	0.00000000	-0.87335534
H	-1.17574682	0.00000000	-1.15932281
H	0.58787341	1.01822661	-1.15932281
H	0.58787341	-1.01822661	-1.15932281
